# Novel {Pb_6_} wheels based zeolite‐type metal‐organic framework for wide temperature range and high sensitivity of luminescent thermometer

**DOI:** 10.1002/smo2.70086

**Published:** 2026-07-28

**Authors:** Yi‐Na Li, Na Sun, Yun‐Long Wu, Xiao‐Gang Yang, Yang‐Tian Yan, Guo‐Ping Yang, Yao‐Yu Wang

**Affiliations:** ^1^ School of Materials Science & Engineering Xi'an Polytechnic University Xi'an China; ^2^ College of Chemistry and Chemical Engineering Luoyang Normal University Luoyang China; ^3^ College of Chemistry & Materials Science Northwest University Xi'an China

**Keywords:** photoluminescence, relative sensitivity, temperature‐dependent, zeolite‐type MOF

## Abstract

The development of luminescent thermometers synchronously exhibiting high sensitivity and stability in a wide temperature range poses significant challenges. Herein, a novel {Pb_6_} wheels based metal‐organic framework (MOF) formulated as [Pb(pyIPA)]·1.2H_2_O (**Pb‐pyIPA,** H_2_pyIPA = 4‐(pyridin‐4‐yl)isophthalic acid) was synthesized via a solvothermal procedure. The **Pb‐pyIPA** MOF possesses a new (4,4)‐connected zeolite‐type structure with dynamic nanotubes modified by {Pb_6_} wheels. It shows intensive green emission with a long lifetime of 180 ns and maintains 98.5% of its initial emission intensity after immersion in water for 90 days. By contrast, temperature‐dependent photoluminescence explorations demonstrate that the emission intensity and lifetime decreased linearly over the temperature range of 298–423 K, achieving a maximal thermal quenching of 93%, relative sensitivity (S_r_) of 1.03% K^−1^ (intensity‐based) and 0.97% K^−1^ (lifetime‐based). These values surpass those of most reported MOFs and commercial inorganic phosphors. Mechanism of high temperature sensitivity was explored by the combination of single crystal X‐ray diffraction analysis and density functional theory calculations. The diameter of {Pb_6_} wheel can be reversibly tuned under the stimulation of external temperature (298–400 K), leading to a significant decrease in the electron cloud distribution around the {Pb_6_} wheels. Thus, both the emission intensity and lifetime can be vastly affected by the dynamic structural transformation. In addition, the stability and reusability of **Pb‐pyIPA** were also investigated, which can maintain high sensitivity under reversible temperature variations between 298 and 423 K for 5 cycles. This study offers a new perspective for balancing the contradiction between high sensitivity and stability of luminescent thermometers in a wide operational temperature range.

## Graphical abstract

The expansion of {Pb_6_} wheels in zeolite‐type MOFs led to emission thermal quenching of 93%, relative sensitivity of 1.03% K^−1^ (intensity‐based) and 0.97% K^−1^ (lifetime‐based) in a wide temperature range of 298–423 K.

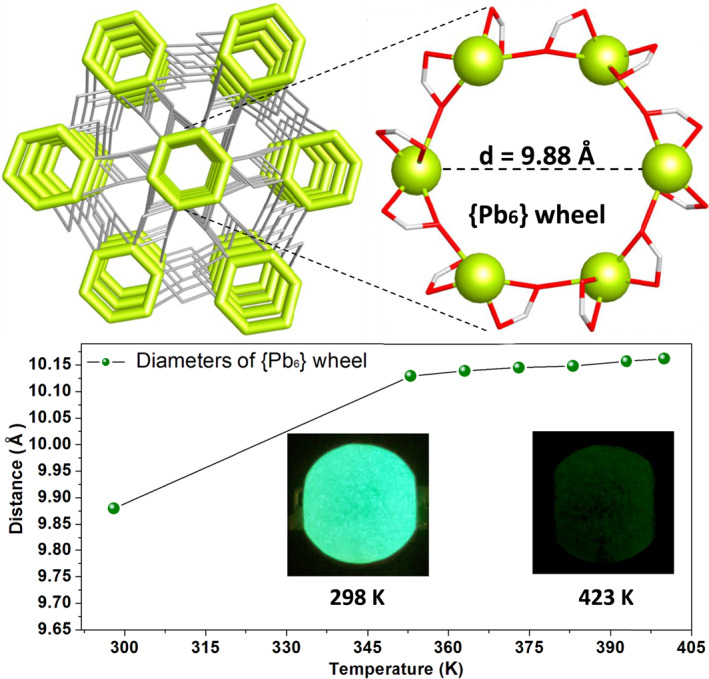

## 1 INTRODUCTION

Luminescent thermometers are a kind of optical sensor that monitor parameters of emission intensity, lifetime, peak position and intensity ratio based on the temperature‐sensitive characteristics of luminescent materials. In recent years, due to their advantages such as non‐contact measurement, and electromagnetic interference resistance, which have been widely applied in biomedical fields, industrial monitoring, micro/nano devices, and other domains.[^1^, ^2^] The prototype of luminescent thermometers can be traced back to studies on rare‐earth‐doped phosphors. For instance, the temperature dependence of luminescent lifetime in rare‐earth ions (Eu^3+^, Tb^3+^) was discovered.[^3^, ^4^] Nonetheless, hampered by limitations in material synthesis and detection technologies, such systems exhibited relatively low thermal sensitivity, notwithstanding their broad operating temperature window. The core reason for the low sensitivity lies in the limitations of material synthesis techniques. Specifically: (1) Non‐uniform doping in solid‐state synthesized phosphors leads to inhomogeneous ion distribution, resulting in unstable luminescence signals[^5^, ^6^]; (2) Matrix defects in hosts introduce lattice imperfections that quench fluorescence and reduce signal‐to‐noise ratios[^7^, ^8^]; (3) Encapsulation of guest molecules (such as rhodamine, inorganic ions) causes rapid photo bleaching under high temperatures or illumination, significantly shortening the operational lifetimes of the optical materials. By contrast, organic optical functional materials exhibit high temperature sensitivity but are limited in a narrow operating temperature range. For instance, AIEgen‐based thermometers perform high sensitivity in the physiological temperature range (30–45°C) for biomedical applications but lose sensitivity at temperatures above 100°C due to thermal decomposition.^[2]^ Therefore, the development of novel luminescent thermometers that simultaneously exhibit a broad operating temperature range and high thermal sensitivity still constitutes a formidable challenge.[^9^, ^10^]

Recently, metal‐organic frameworks (MOFs) are porous crystalline materials formed by the self‐assembly process of metal ions/clusters and organic ligands via coordination bonds under the solvent thermal synthesis methods, combining the stability of inorganic materials with the functional tunability of organic components.[^11^, ^12^, ^13^, ^14^] Different from the weak electrostatic attraction and intermolecular interaction in organic‐inorganic hybrid photosensitive materials, strong coordination bonds between organic ligands and metal ions would enable physicochemical stability for MOF materials. Metal‐organic frameworks have demonstrated significant potential in luminescent temperature sensing owing to their engineerable structures, high specific surface areas, and tunable luminescent properties.[^15^, ^16^, ^17^] The common luminescent temperature sensing mechanisms are as follows: Firstly, thermal quenching of ligand fluorescence: Elevated temperatures accelerate non‐radiative transitions, thereby reducing the luminescent intensity of organic ligands[^18^, ^19^]; Secondly, lifetimes of the luminescent center metal ions (e.g., Eu^3+^, Tb^3+^) exhibit significant temperature sensitivity, while the rigid framework of MOFs shields them from environmental quenching^[20]^; Thirdly, modulation of host‐guest energy transfer: When different molecules are encapsulated within MOF pores, adsorbed solvent or gas molecules undergo temperature‐induced changes that can modulate energy transfer efficiency, consequently altering luminescence properties.^[21]^ MOFs exhibit unique advantages in luminescent temperature sensing owing to the precisely controllable chemical composition and highly tunable structural design, which can enable customized developments over a wide temperature ranges and complex environmental requirements.[^22^, ^23^] However, the inadequate hydrolytic and thermochemical stability of MOFs significantly impedes their practical applications.[^24^, ^25^] In the future, MOFs with a stable structure, as luminescent thermometer sensors, are expected to achieve wider applications in fields such as biomedicine, industrial monitoring, and extreme environments through stability optimization and intelligent design.

Based on the discussion above and in conjunction with the prior research on MOFs for temperature sensing, the selection of central metal ions and organic linkers plays a decisive role in constructing targeted MOFs with desired structures and properties. In this study, a rigid nitrogen‐ and oxygen‐containing organic ligand 4‐(pyridin‐4‐yl)isophthalic acid (H_2_pyIPA) was chosen as the building linker and the 5d^10^ electronic configuration Pb^2+^ ion was selected as the center metal ion to construct the MOF. A novel zeolite‐type MOF [Pb(pyIPA)]·1.2H_2_O (Pb‐pyIPA) was obtained via the solvothermal synthesis procedure. Structural analysis revealed that the framework features a new type of zeolite‐type structure, containing one‐dimensional channels with wheel‐like {Pb_6_} cluster as window. The Pb‐pyIPA MOF emits intensive green light with a long lifetime of 180 ns. After being immersed in water for 3 months, it still retains 98.5% of its initial emission intensity. The temperature‐dependent measurements afforded a maximal thermal quenching of 93%, maximum relative sensitivity (Sr) of 1.03% K^−1^ (intensity‐based) and 0.97% K^−1^ (lifetime‐based) in a broad temperature range of 298−423K. Experimental and theoretical calculations were combined to investigate the optic properties and sensing mechanism. The results indicate that Pb‐pyIPA can serve as a promising intensity‐ and lifetime‐dependent luminescent thermometer applicable over a broad temperature range.

## 2 RESULTS AND DISCUSSION

Colorless rod‐like crystals for single crystal X‐ray diffraction analysis were synthesized under solvothermal conditions at 105°C for 72 h using Pb(NO_3_)_2_ and H_2_pyIPA (Supporting Information S1; Figure S1). The as synthesized sample was characterized by the powder X‐ray diffraction. The results show that the main diffraction peaks of the as synthesized samples consist well with the peaks simulated from the single crystal diffraction data (Supporting Information S1; Figure S2), confirming the successful preparation of a pure phase. Single‐crystal X‐ray diffraction analysis revealed that Pb‐pyIPA crystallizes in the hexagonal crystal system with space group *R*‐3, the asymmetry unit of which consisted of one Pb(II) ion, one fully deprotonated organic ligand pyIPA^2‐^, and 1.2 lattice aqueous molecule. As shown in Supporting Information S1; Figure S3a, the central Pb(II) ion adopts a six‐coordinate geometry, ligated by five oxygen atoms from three carboxylic groups of three independent pyIPA ligands and one nitrogen atoms from another independent pyIPA ligand, resulting in a distorted octahedron geometric configuration. The selected bonds and angles for **Pb‐pyIPA** have been added Supporting Information S1; Table S2.

**FIGURE 1:**
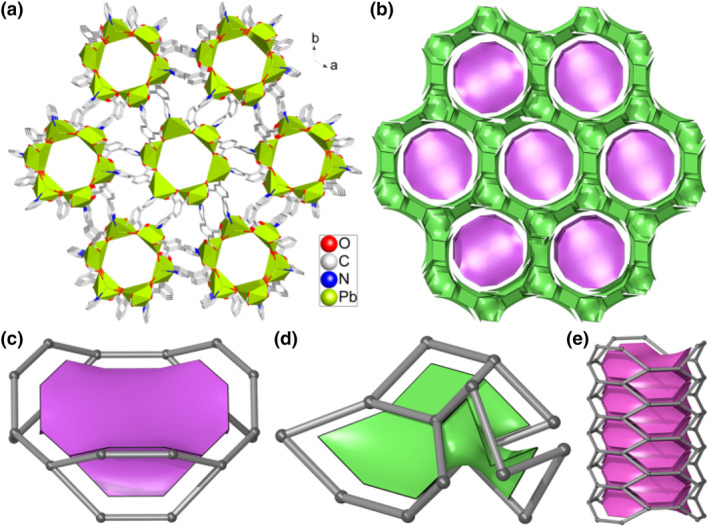
The structure information for **1**: (a) View of the 3D network of **Pb‐pyIPA** along *c* direction. (b) Nature tiling representation of the (4, 4)‐connected net. View of [6^3^·12^2^] (c) and [4·6·8^3^] (d) tiles. (e) Tiling view of 12‐number ring channel in **Pb‐pyIPA**.

In **Pb‐pyIPA**, each pyIPA ligand adopts a μ_6_‐bridging mode to link four Pb(II) centers through a chelating carboxylate (μ_1_–η^1^:η^1^), a chelate‐monodentate carboxylate (μ_2_–η^1^: η^2^) and a monodentate pyridine group (Supporting Information S1; Figure S3b). The adjacent Pb(II) centers are connected by pyIPA to form a {Pb_6_} wheel (Supporting Information S1; Figure S4). These {Pb_6_} units are further connected by the pyIPA ligands to give a 3D porous framework with the channels modified by {Pb_6_} wheels and occupied by guest water molecules (Figure 1a and Supporting Information S1; Figure S5). To fit the geometric requirements, the flexible pyIPA ligands were forced to exhibit a twisty conformation with a torsion angle of 56.19° between the pyridine and benzene rings (Supporting Information S1; Figure S6). The μ_2_–η^1^: η^2^ carboxylate group also deviated from the plane of the benzene ring by 33.52°. Based on such a twisty conformation, 4‐stranded helical chains with the same chirality running along *c* direction can be observed; these intertwining helical chains further generate a triangular channel (Supporting Information S1; Figure S7). In order to give a clear structural description, topological explorations reveal that the center Pb(II) ions and the pyIPA ligands can be regarded as 4‐connected nodes. The whole structure thus can be simplified as a new (4,4)‐connected zeolite‐type topology with the point symbol of [6^3^·12^2^] [4·6·8^3^] (Figure 1b–e and Supporting Information S1; Figures S8 and S9).

The TGA curve of **Pb‐pyIPA** showed two‐step weight losses. The first weight loss of about 5.2% occurs up to approximately 95°C, which is likely attributed to the removal of lattice water molecules. Beyond 380°C, the TGA curve shows a sharp decline (Supporting Information S1; Figure S10a), indicating that **Pb‐pyIPA** possesses high thermal stability, a relatively uncommon characteristic among porous MOF materials. To demonstrate its permanent porosity, the **Pb‐pyIPA** was directly activated at 100°C for 4 h to produce the desolvated material. The successful activation was confirmed by TGA and PXRD patterns (Supporting Information S1; Figures S10b and S11). The TGA curve of desolvated **Pb‐pyIPA** shows a single‐step weight loss and can also retain its microstructure up to about 380°C, consistent with the removal of residual lattice water. The N_2_ adsorption isotherm was investigated at 77 K, which exhibited a type‐I gas adsorption curve with a saturated adsorption of 201.4 cm^3^ g^−1^. From the N_2_ adsorption data, the BET surface area was determined to be 772.5 cm^3^ g^−1^ and the langmuir surface area to be 1662.5 cm^3^ g^−1^ (Supporting Information S1; Figure S12a). Furthermore, the water‐storage capacity was evaluated through water vapor adsorption experiments. The desolvated **Pb‐pyIPA** shows a high water adsorption capacity of 120 cm^3^ g^−1^ at a low relative pressure of 0.1 *P*/*P*_0_ at 298 K. The water uptake increased to 229 cm^3^ g^−1^ at a high pressure of 1 *P*/*P*_0_ at 298 K (Supporting Information S1; Figure S12b), which is comparable to or even exceeds that of typical MOFs. The combination of high water‐uptake capacity and excellent stability suggests that the desolvated **Pb‐pyIPA** could serve as a potential water‐storage material.[^26^, ^27^]

**FIGURE 2:**
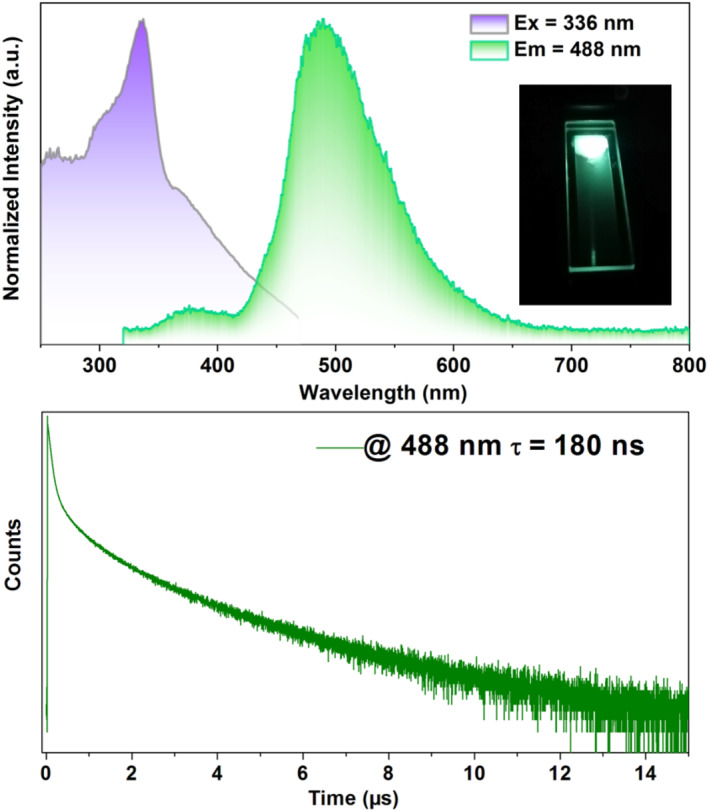
The excitation, emission spectra and lifetime for **Pb‐pyIPA**: (a) Normalized excitation, emission spectra (Insert shows the powder sample of desolvated **Pb‐pyIPA** under 336 nm ultraviolet (UV) light); (b) photoluminescence decay curve of **Pb‐pyIPA** in solid state measured at room temperature.

Metal‐organic frameworks based on Pb(II) ions with d^10^ electron configuration and conjugated organic linkers have attracted considerable interest from chemists due to their various applications in photochemistry, chemical sensors, light emitting diodes and so on.[^28^, ^29^, ^30^] Accordingly, the photophysical properties of the solid‐state sample **Pb‐pyIPA** were systematically investigated at room temperature. As depicted in Figure 2a, the excitation spectrum shows a broad area from 250 to 480 nm, with a maximum peak at 336 nm. Under excitation at 336 nm, the emission spectrum displays a maximum peak at 488 nm. The solid state sample emits intense green light under ultraviolet (UV) irradiation (336 nm) (insert in Figure 2a). Photoluminescence (PL) decay curve measured at room temperature revealed a long emission lifetime of 180 ns (Figure 2b). The single‐exponential decay profile and the prolonged emission lifetime indicate the charge‐transfer characteristics in **Pb‐pyIPA**. This emission lifetime is notably longer than that of typical organic‐inorganic hybrid perovskite materials.[^31^, ^32^] The long lifetime can be assigned to the heave atom effect of Pb(II) ions, which can enhance spin orbital coupling and promote efficient intersystem crossing from singlet excited state to triplet state.^[33]^

**FIGURE 3:**
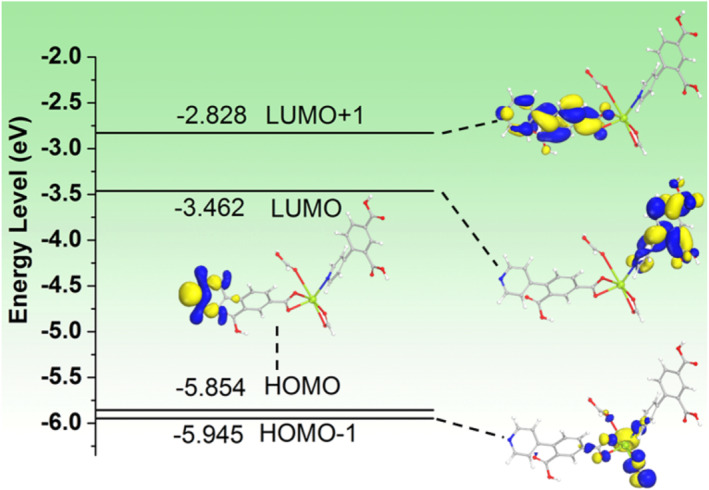
View of the HOMOs, LUMOs and relevant energy level/gap for the density functional theory (DFT) optimized structure of **Pb‐pyIPA**.

To gain insight into the PL mechanism of **Pb‐pyIPA**, frontier molecular orbitals were investigated via density functional theory calculations. As shown in Figure 3, a clear separation is observed between the highest occupied molecular orbitals (HOMO‐1) and the lowest unoccupied molecular orbitals (LUMO). This result observation aligns with the behavior reported for organic‐inorganic hybrids and AD‐based cocrystal systems.[^34^, ^35^] The electron density of HOMO‐1 is almost entirely localized on the central Pb(II) ion, whereas the electron density of HOMO, LUMO and LUMO+1 is fully distributed on the organic ligand. Therefore, the electronic transition from the HOMO‐1 to LUMO corresponds to charge transfer from the Pb(II) ion center to the pyIPA ligand. The energy levels of HOMO‐1 and LUMO are −5.945 eV and −3.462 eV, respectively. The energy gap between HOMO‐1 and LUMO is calculated to be 2.483 eV, which matches well with the PL emission peak observed at around 488 nm (2.54 eV). Thus, the above mentioned photophysical behavior involves an MLCT process from the Pb(II) ion to the organic links, which usually affords a prolonged excitation lifetime.

**FIGURE 4:**
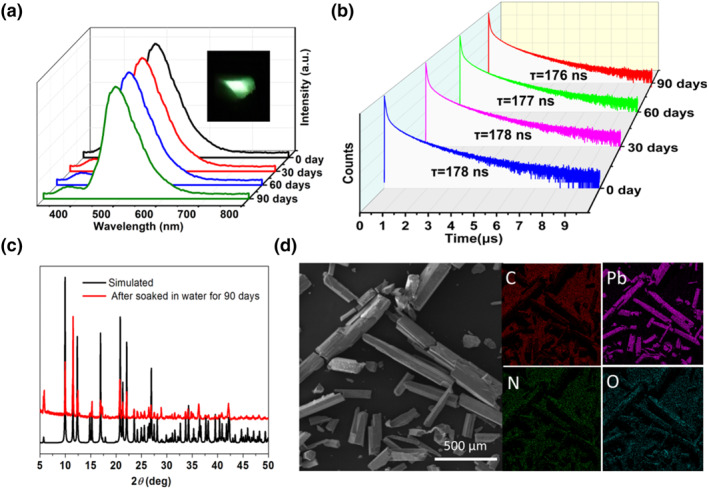
The environmental stability properties of **Pb‐pyIPA**: (a) Emission spectra for **Pb‐pyIPA** after immersing in aqueous solution for different times (Insert shows the recollected sample radiated under 336 nm light); (b) PL decay curves of **Pb‐pyIPA** after the different water‐soaking times; (c) PXRD pattern and (d) SEM and elemental mapping images of **Pb‐pyIPA** after soaking in water for 90 days.

In terms of real‐world practical applications, water stability represents a critical factor that must be taken into account. To assess its water stability, 100 mg of powdered sample of **Pb‐pyIPA** was placed in 20 mL of deionized water at room temperature and maintained for 90 days. As depicted in Figure 4a and Supporting Information S1; Figure S13, the recollected sample also exhibits a main emission peak at 488 nm and maintains 98.5% of its initial emission intensity. As determined from the PL decay curves of different samples after immersion in water for various days, the pristine raw sample remains a long lifetime of 178 ns (Figure 4b). While the PL decay curve of the sample after immersing in water for 90 days still exhibits a PL lifetime of 176 ns (Figure 4b). The experimental results demonstrate the robust optical stability of the sample upon prolonged aqueous immersion. Good agreement between the experimental PXRD pattern and the simulated confirms the retention of the crystal structure in the recollected sample after water stability tests (Figure 4c). SEM images (Figure 4d and Supporting Information S1; Figure S14) demonstrate no obvious morphological changes. To date, water stability is still a thorny problem for organic–inorganic hybrid materials, although these materials possess excellent optical performance. Most of the reported works are focused on the surface modification.[^31^, ^36^, ^37^, ^38^] By contrast, Pb(II) based MOF in this work possesses high water stability without any treatment. This can result from the formation of bulk {Pb_6_} wheel, which can anchor more flexible pyIPA ligands (Supporting Information S1; Figure S15) to form a stable network to prevent corrosion from water molecules.

**FIGURE 5:**
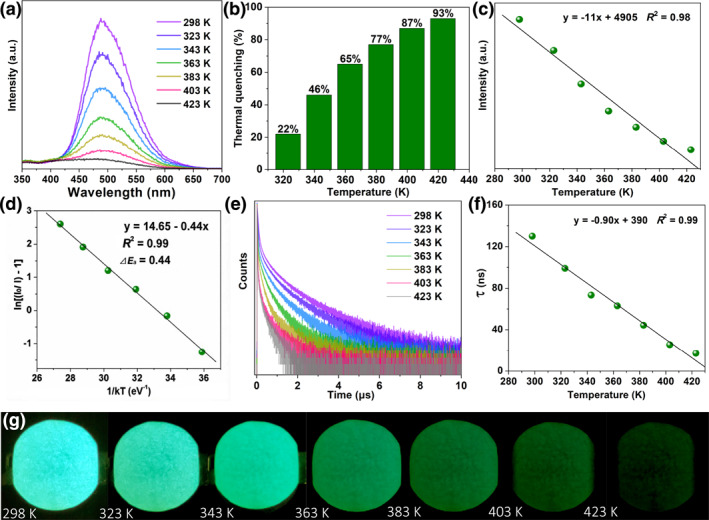
The PL properties of **Pb‐pyIPA**: (a) Temperature‐dependent emission spectra of desolvated **Pb‐pyIPA**. (b) Histograms showing the thermal quenching emission intensity values compared to room temperature (298 K). (c) Temperature‐dependent maximum PL intensity and fitted curve. (d) Relationship of ln[(I_0_/I_T_) − 1] versus 1/kT activation energy graph. (e) Temperature‐dependent decay traces. (f) Temperature‐dependent decay time and fitted curve. (g) Photographs of desolvated **Pb‐pyIPA** under ultraviolet lamp irradiation from 298 to 423 K. All above data were recorded in the temperature range of 298–423 K (excited at 336 nm).

Luminescent thermometers in inorganic and organic optical materials have been reported for decades and have received intense interest in the recent years.[^39^, ^40^, ^41^] However, the high sensitivity but narrow temperature range of organic materials, along with the low sensitivity but the wide temperature range of inorganic materials, have limited their application in luminescent temperature sensors. Optical materials that simultaneously possess high sensitivity and a broad temperature‐sensing range are rarely reported. As a kind of inorganic‐organic hybrid material, the optical properties of desolvated **Pb‐pyIPA** were systematically investigated over a wide temperature range of 298–423 K. As shown in Figure 5a, temperature‐dependent PL measurements indicate that the emission intensity decreases sharply by 65% of its initial value as the temperature increases from 298 to 363 K, demonstrating remarkably high sensitivity compared with reported optical materials.[^42^, ^43^, ^44^] Upon further increasing the temperature to 423 K, the emission intensity gradually declines by a total of 93% (Figure 5b). Meanwhile, the emission peak position remains essentially unchanged at different temperatures. A linear relationship between the maximum PL intensity and temperature in the range of 298–423 K can be fitted with the function of *y* = −11*x* + 4905, yielding a correlation coefficient of 0.98 (Figure 5c). These results confirm that the emission intensity of **Pb‐pyIPA** is highly sensitive to temperature, suggesting its potential as an intensity based luminescent thermometer. Relative sensitivity (S_r_) is a key parameter for evaluating the temperature‐sensing performance. Based on the maximum emission intensities at different temperatures, the emission intensity based S_r_ was calculated to be 0.75%–1.03% K^−1^ over the range of 298–423 K, with a maximum value of 1.03% K^−1^ at 70°C (Supporting Information S1; Figure S16a). This high S_r_ is comparable to or even exceeds that of some reported inorganic‐organic hybrid optical temperature sensing materials, and surpasses the performance of most MOF‐based thermometers reported to date (Supporting Information S1; Table S3). To further quantitatively characterize the degree of luminescent thermal quenching and elucidate its underlying microscopic mechanism, the activation energy (Δ*E*_*a*_) was calculated from the Arrhenius equation: *I*_*T*_ = *I*_0_/[1 + *A* exp(−Δ*E*_*a*_/*kT*)], where *I*_*0*_ and *I*_*T*_ are the luminescence intensities at room temperature and different test temperatures, respectively. A is a constant, T is the absolute temperature (K), and *k* is the Boltzmann constant (8.629 × 10^−5^ eV K^−1^).^[45]^ A higher Δ*E*_*a*_ leads to a more intense thermal quenching effect, resulting in a more pronounced luminescent thermal quenching of the material at high temperatures. From the slope of the fitting line in the plot of ln[(*I*_*0*_/*I*_*T*_) − 1] versus 1/kT (Figure 5d), it gives rise to a Δ*E*_*a*_ of 0.44 eV, which can be assigned to the thermal movement of ions in crystals, leading to lattice distortion and destroying the energy barrier of the luminescence center.

Long luminescent lifetime‐based temperature sensing is engineered around the strong coupling response between luminescent lifetime and temperature, along with superior anti‐interference performance and high detection accuracy. It not only mitigates the critical limitations of conventional short‐lifetime fluorescent temperature sensing (e.g., detection blind spots and inadequate anti‐interference capability) but also exhibits distinctive technical merits in non‐contact precise temperature measurement, complex environment monitoring, and high spatiotemporal resolution detection. Investigation of lifetime‐based thermometry shows that the decay kinetics of **Pb‐pyIPA** also displays significant temperature dependence. As presented in Figure 5e, the lifetime decay traces were recorded over the temperature range of 298–423 K. As the temperature rises, the decay time decreases sharply and exhibits a lifetime‐based thermal quenching. The average decay time and temperature follow a linear relationship fitted by *y* = −0.9*x* + 390, with a correlation coefficient of 0.99 (Figure 5f). These results indicate that the lifetime of **Pb‐pyIPA** is sensitive to temperature, demonstrating it can function as a lifetime dependent luminescent thermometer‒a rare feature among MOF based thermometers. Additionally, the lifetime based S_r_ was evaluated in the range of 0.69–0.97% K^−1^ over 298–423 K, reaching a maximum 0.97% K^−1^ at 343 K (Supporting Information S1; Figure S16b). This high S_r_ value is comparable to, or even superior to that of some reported inorganic‐organic hybrid optical temperature sensing materials, and it surpasses most MOF‐based thermochromic systems documented so far (Supporting Information S1; Table S4). Moreover, cyclic tests on luminescence lifetime reveal that the PL decay characteristics of **Pb‐pyIPA** are well preserved after repeated cycling operations, demonstrating the potential of the complex for reusable temperature sensing applications (Supporting Information S1; Figure S17). With its high sensitivity, high recyclability and broad temperature range, **Pb‐pyIPA** addresses the shortcomings of existing inorganic and organic temperature sensors, positioning it as a promising candidate for practical luminescent thermometers. To further probe its optical characteristics, photographs of the sample under ultraviolet irradiation were taken from 298 to 423 K. As shown in Figure 5g, the sample emits bright green light at 298 K, but appears dark after being heated to 423 K, illustrating a clear temperature‐dependent emission quenching. Taken together, these studies demonstrate that **Pb‐pyIPA** serves as a sensitive dual function thermometer that operates reliably through both PL intensity and lifetime measurements. Further, the recyclability of the sensor was experimentally investigated. Cycling tests revealed that its emission intensity showed negligible decay upon returning to room temperature after 5 consecutive heating–cooling cycles between 298 and 423 K (Supporting Information S1; Figure S20). Its temperature sensing sensitivity also exhibits no significant variation. Meanwhile, the PXRD pattern after cycling measurements consists well with the pattern simulated from the crystal data (Supporting Information S1; Figure S19). The SEM image presented in Supporting Information S1; Figure S18 confirms that no distinct morphological variations can be identified. All these results demonstrate the excellent reversibility, fatigue resistance, and cycling stability of **Pb‐pyIPA** for practical temperature‐sensing applications.

**FIGURE 6:**
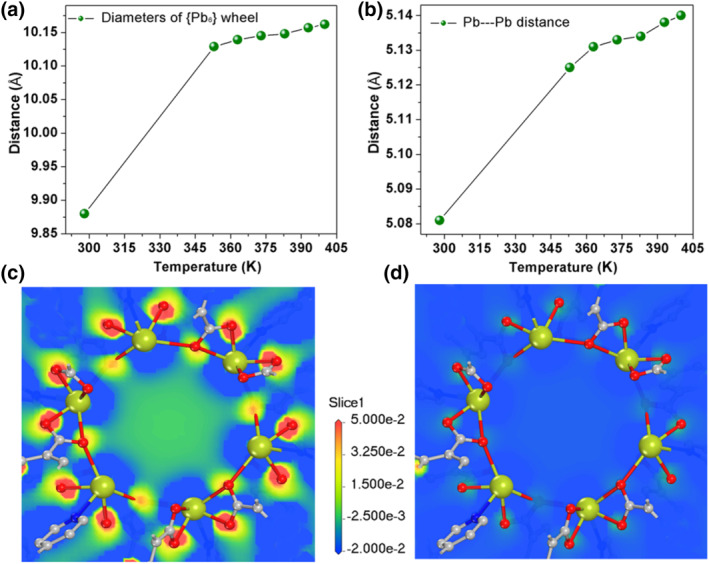
The network dynamics information and charge density distribution for **Pb‒pyIPA**: The diameters of {Pb_6_} wheel (a) and Pb···Pb distance in each {Pb_6_} wheel (b) increased in the temperature range of 298–400 K. The charge density distribution maps simulated at 298 (c) and 400 K (d).

To gain insight into the high‐performance thermal sensing mechanism of **Pb**‒**pyIPA**, systematic investigations including detailed single‐crystal X‐ray diffraction and theoretical calculations were carried out. As the high thermal stability of **Pb‐pyIPA** was proven by the TGA curve, the single crystal X‐ray diffractions were measured at different temperatures (353 K, 363 K, 373 K, 383 K, 393 K, 400 K). The single crystal data at different temperatures reveal that the crystal structure of **Pb‐pyIPA** has undergone great changes although it still retains the original hexagonal crystal system, *R*‐3 space group and topology to that of 298 K. Firstly, the difference lies in subtle variations in the unit cell parameters. The unit cell parameters exhibit an increasing trend with rising temperature (Supporting Information S1; Tables S1 and S5). The volume increases from 6083.9 to 6154.4(2) Å^3^. Correspondingly, *a*/*b* and *c* changed in the range of 30.759–30.881 Å, 7.425–7.452 Å, respectively. As the window of the channels in **Pb‐pyIPA**, the diameter of {Pb_6_} wheel also exhibited an increasing trend from 9.880 Å to 10.162 Å (Figure 6a and Supporting Information S1; Figure S21) as the temperature rose from 298 to 400 K. Additionally, the Pb···Pb distance in each {Pb_6_} wheel increased from 5.081 to 5.140 Å (Figure 6b). Meanwhile, the dihedral angles between the benzene ring and the pyridine ring of the pyIPA flexible organic ligand displayed an increasing trend (56.265–60.893°) with rising temperature (Supporting Information S1; Figure S22).

The charge density distribution map simulated at 298 K clearly reveals the electronic distribution characteristics within the {Pb_6_} wheel, indicating that dense and highly localized electronic charges are concentrated around the lead metal clusters and their coordination environments (Figure 6c). This distinct charge accumulation vividly reflects the significant electronic interactions and charge transfer between the Pb(II) centers and the pyIPA ligands, providing crucial theoretical support for further understanding the electronic structure, luminescence mechanism, and thermal‐responsive behavior of the material. By sharp contrast, with increasing temperature up to 400 K, **Pb‐pyIPA** undergoes obvious thermal expansion, leading to the enlargement of the unit cell volume and the increase of the Pb···Pb distance. Such thermally induced structural relaxation and spatial variation effectively weaken the electronic interactions within the metal cluster and between metal centers and ligands (Figure 6d), thereby diminishing the electronic delocalization, coupling effects, and radiative transition characteristics of the metal cluster, which further influence the luminescence behavior and temperature‐responsive performance.[^46^, ^47^]

## 3 CONCLUSION

In summary, a novel 3D porous MOF (**Pb‐pyIPA**) with high aqueous and thermal stability was synthesized via the solvothermal procedure. Structural analysis revealed that there exist two kinds of 1D inerratic nanotubes (cylindrical and triangular nanotube), showing a new (4,4)‐connected topological zeolite‐type structure with the point symbol of [6^3^·12^2^] [4·6·8^3^]. Optical explorations showed that the **Pb‐pyIPA** displays a sharp decrease in emission intensity of 93% relative to its initial intensity in the temperature range of 298–423 K, accompanied by a maximum relative sensitivity (S_r_) of 1.03% K^−1^ (intensity‐based) and 0.97% K^−1^ (lifetime‐based) at 343 K. This performance is comparable to, or even superior to, that of commercial inorganic phosphors. The mechanism for the high sensitivity of the title MOF was exploded by the combination of crystallographic analysis and theoretical calculations: The thermally induced dynamic expansion of the flexible wheel‐like structure reduces the electron density around the {Pb_6_} cluster, thereby lowering the electronic delocalization, coupling effects, and radiative transition characteristics, leading to significant emission quenching. Therefore, this work not only provides a reference for designing and synthesizing metal cluster based MOF with flexible characteristics but also addresses the contradiction between the stability and sensitivity of luminescent thermometers in a wide temperature range.

## CONFLICT OF INTEREST STATEMENT

The authors declare no conflicts of interest.

## ETHICS STATEMENT

This study did not involve human participants, animals, or any sensitive biological data. No ethical approval or informed consent is required.

## Supporting information

Supporting Information S1

## Data Availability

The data that support the findings of this study are available on request from the corresponding author. The data are not publicly available due to privacy or ethical restrictions.
