# Supplementary material for: Novel {Pb6} wheels based zeolite‐type metal‐organic framework for wide temperature range and high sensitivity of luminescent thermometer
Source: Smart Mol. 2026 Jul 28:e70086. Online ahead of print. doi: 10.1002/smo2.70086 (PMC13415905; doi:10.1002/smo2.70086)
Supplement: Supplementary file 1 — Supporting Information S1 [file SMO2-9999-0-s001.docx]

Supporting Information

Novel {Pb_6_} wheels based zeolite-type MOF for wide temperature range and high sensitivity of luminescent thermometer

Yi-Na Li^a#^, Na Sun^a#^, Yun-Long Wu^a^*, Xiao-Gang Yang^b^*, Yang-Tian Yan^a^, Guo-Ping Yang^c^* and Yao-Yu Wang^c^

**1. Experimental Section**

**1.1. Materials and methods**

All the starting reagents and solvents were commercially available and used as received without further purification. Powder X-ray diffraction (PXRD) patterns were measured by using the Bruker D8-ADVANCE X-ray diffractometer with Cu *Kα* radiation (λ = 1.5418 Å). The IR spectrum was recorded in the range of 4000–400 cm^-1^ on a Nicolet 6700 (Thermo) FT-IR spectrometer with KBr pellets. Thermogravimetric analyses (TGA) were determined with a NETZSCH STA 449C microanalyzer under a nitrogen stream at a heating rate of 5 °C min^-1^. Gas and water vapor sorption isotherms were recorded by a Micromeritics 3Flex surface characterization analyzer and a Microtrac Belsorp Max II gas adsorption analyzer. Photoluminescence (PL) spectra and decay curve were tested on Edinburgh FLS1000 fluorescence spectrometer. The temperature dependence PL spectra were measured using a temperature controller attached to a cryostat (Oxford Ltd. Optistat DN2) using an FLS1000 fluorescence spectrometer.

**1.2 Synthesis of** **[Pb(pyIPA)]·1.2H_2_O (Pb-pyIPA)**

Pb(NO_3_)_2_ (0.1 mmol, 33.1 mg) and H_2_pyIPA (0.1 mmol, 24.3 mg) were accurately weighed and placed in a beaker, and 6 mL of DMA and 2 mL of deionized water were injected into the mixed solvent system. After sufficient stirring, the solution system is homogeneous, and then transferred to a 15 ml PTFE-lined stainless steel reactor and placed in an electric blast drying oven. The reaction system was linearly heated to 105 °C for 6 hours, then kept at a constant temperature for 72 hours, and after natural cooling to room temperature, colorless transparent needle-like crystals were observed, FT-IR (cm^-1^): 3420(m), 1694(m), 1596(m), 1543(s), 1355(s), 1301(m), 1087(w), 998(w), 909(w), 827(m), 757(m), 560(w).

**1.3 Dehydration of [Pb(pyIPA)]∙1.2H_2_O (Pb-pyIPA)**

The dehydrated sample was obtained via the vacuum heat treatment of the as synthesized **Pb-pyIPA** at 100 °C for 4 hours. The structure of dehydrated **Pb-pyIPA** has been identified by the single x-ray diffraction and the PXRD.

**1.4 Crystal structure determination**

Temperature dependent single-crystal X-ray diffraction data for dehydrated **Pb-pyIPA** was obtained at 293 K, 353 K, 363 K, 373 K, 383 K, 393 K and 400 K on an Oxford Diffraction SuperNova area-detector diffractometer using mirror optics monochromated Mo Kα radiation (λ = 0.71073 Å). The diffraction data were corrected for Lorentz and polarization effects as well as for empirical absorption based on a multi-scan. The crystal structures of dehydrated **Pb-pyIPA** were solved by direct methods using SHELXS-2014^[1]^ and least-squares refined with SHELXL-2014.^[2]^ Reflection data were corrected using the program SADABS.^[3]^ Anisotropic thermal parameters were applied to non-hydrogen atoms, and all hydrogen atoms from the organic ligands were calculated and added at idealized positions. Other details of relevant crystallographic data are given in Table S1. Selected bond lengths and angles are listed in Table S2. The CIF file of **Pb-pyIPA** (CCDC No. 2480253) can be acquired free of charge from the Cambridge Crystallographic Date Centre via [http://www.ccdc.cam. ac.uk/conts/retrieving.html.](http://www.ccdc.cam.ac.uk/conts/retrieving.html.)

**1.5 Electronic structure calculations**

The density functional theory (DFT) was carried out using Dmol3^[4]^ and CASTEP^[5]^ module in Material Studio software package,^[6]^ respectively. All calculations were performed based on the crystallographic information file (cif) from the single-crystal structure of Pb-pyIPA. The initial geometrical optimizations of the ground state was carried out by Perdew-Wang (PW91) generalized gradient approximation (GGA) method with the double numerical basis sets plus polarization function (DNP). The self-consistent field (SCF) converged criterion was within 1.0 × 10^-5^ hartree atom^-1^ and the converging criterion of the structure optimization was 1.0 × 10^-3^ hartree bohr^-1^. The Brillouin zone is sampled by 1×1×1 k-points, and test calculations reveal that the increase of k-points does not affect the results.

**2. Supporting Figures**





**Figure S1.** Chemical structure of the organic ligand 4-(pyridin-4-yl)isophthalic acid (H_2_pyIPA).


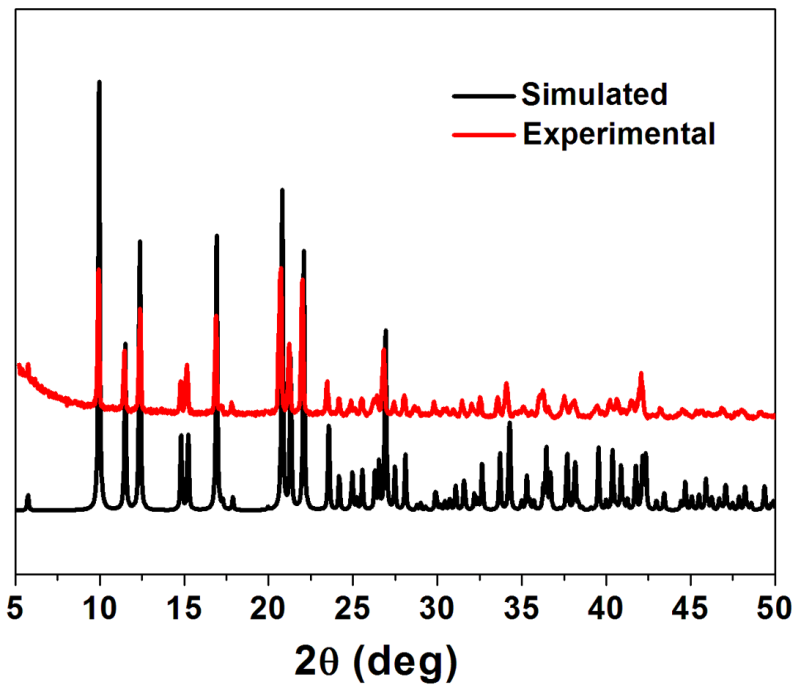


**Figure S2.** Powder X-ray diffraction (PXRD) patterns of simulated (black) and as-prepared (red) [Pb(pyIPA)]·H_2_O (**Pb-pyIPA**) obtained under solvothermal condition.


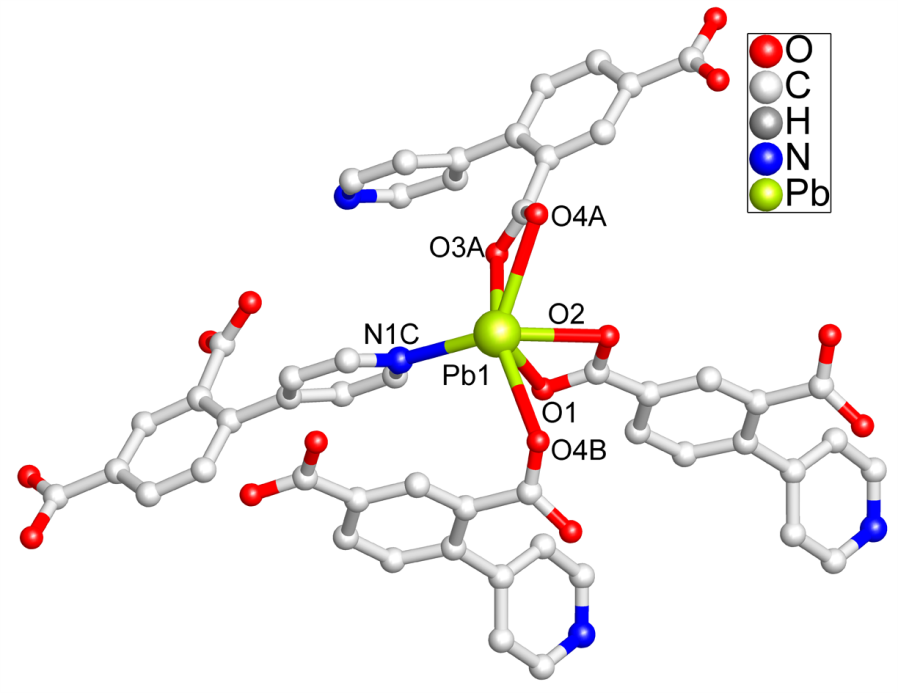


(a)


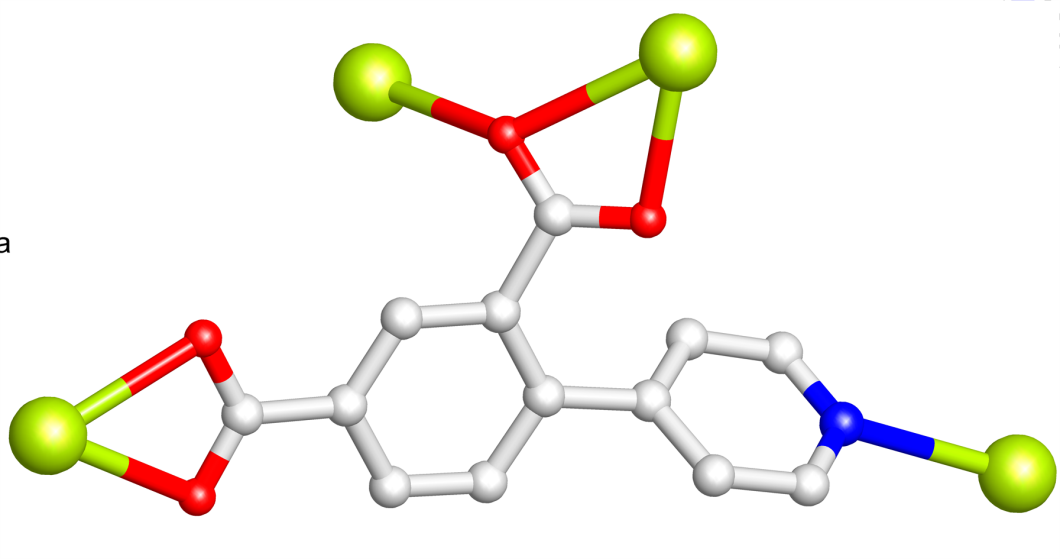


(b)

**Figure S3.** (a) View of local coordination environment of Pb(II) ion in **Pb-pyIPA**. (b) The coordination model of the **pyIPA** ligand.


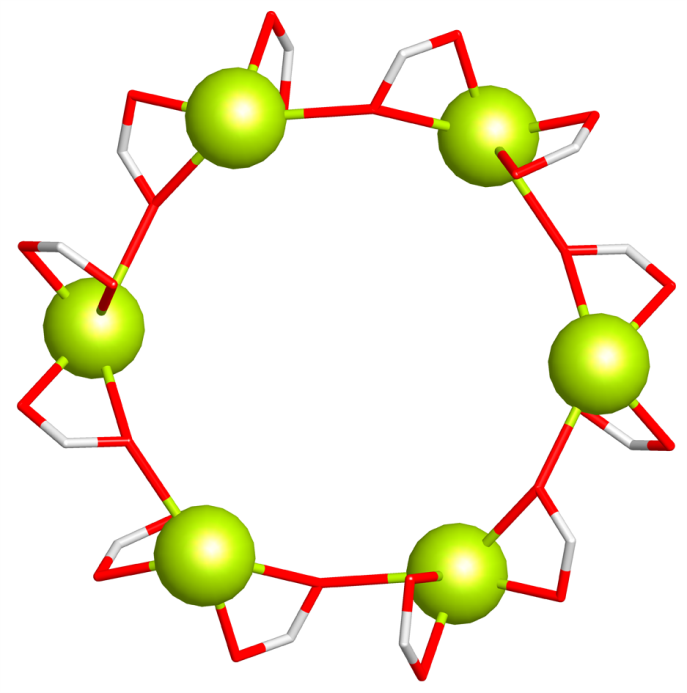


**Figure S4.** View of the {Pb_6_} wheel in **Pb-pyIPA**.


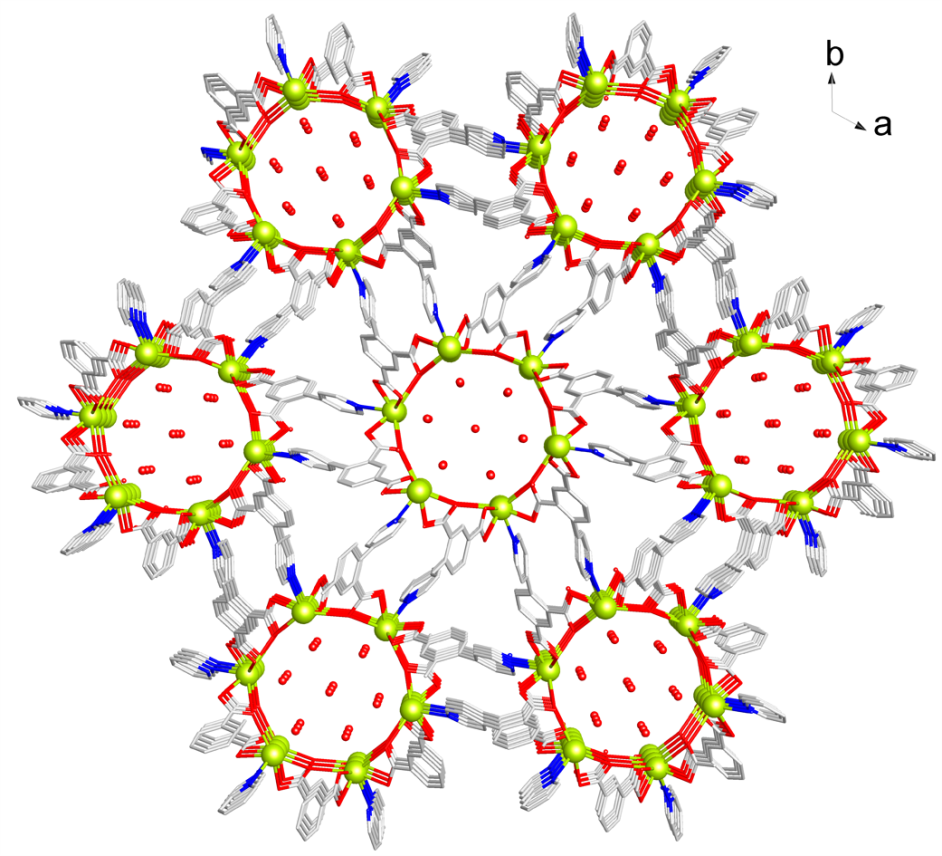


**Figure S5.** View of the 3D network of **Pb-pyIPA** along c direction.


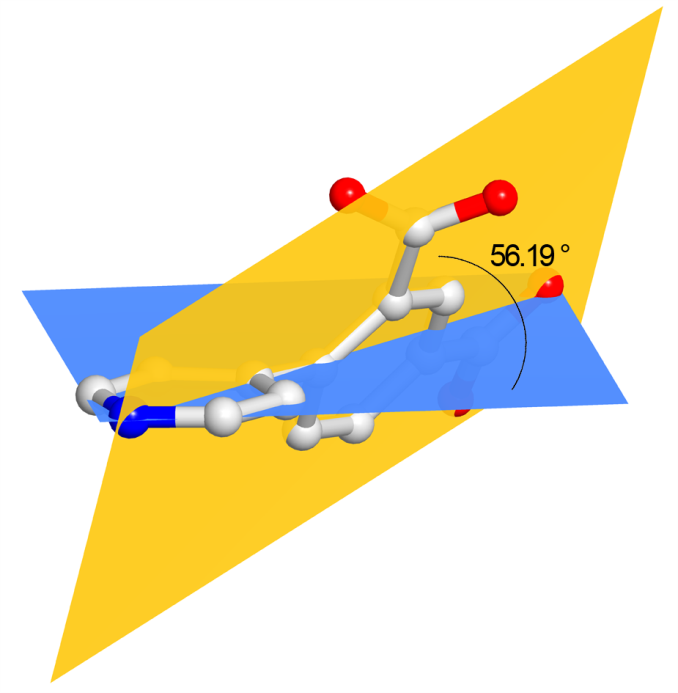


**Figure S6.** The torsion angle between pyridine and benzene ring of pyIPA in **Pb-pyIPA**.


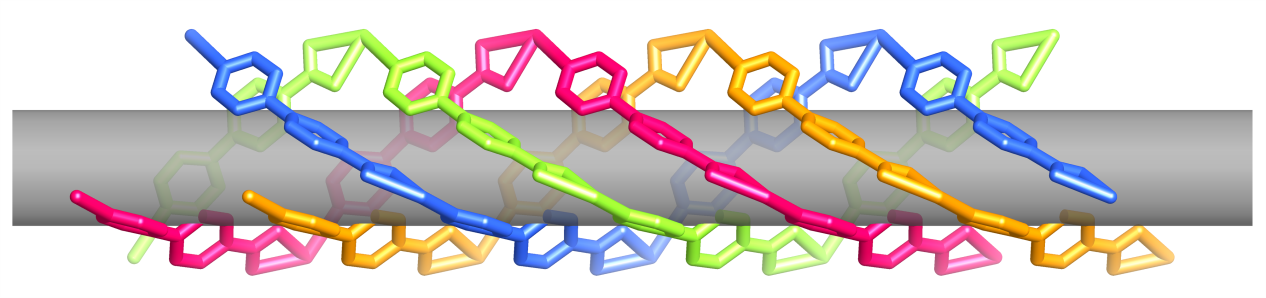


(a)


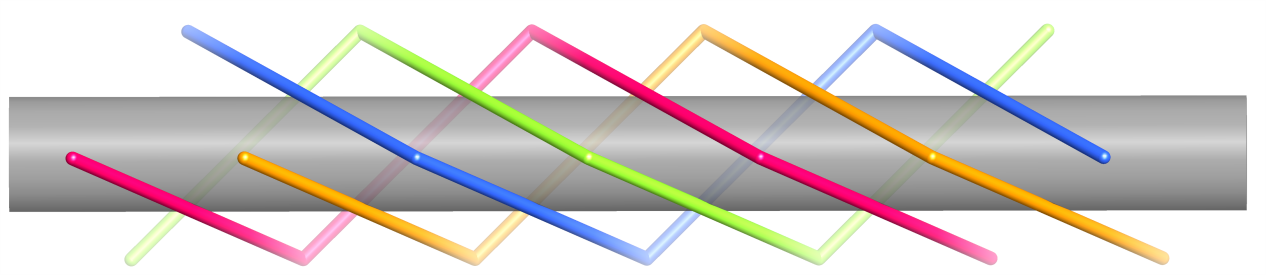


(b)

**
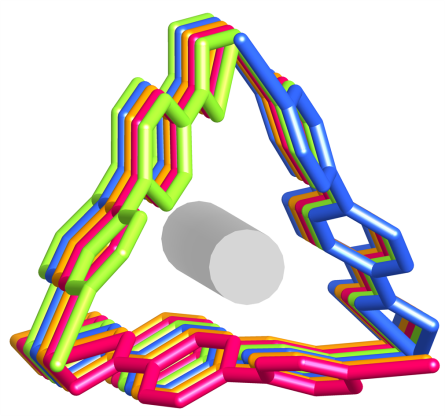

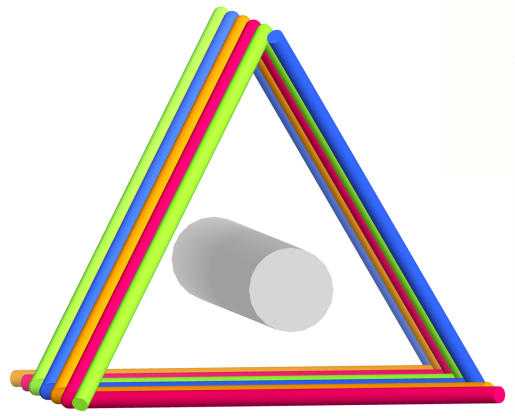
**

(c) (d)

**Figure S7.** Wire and schematic views of 4-stranded helical chains with the same chirality running along c direction.

**
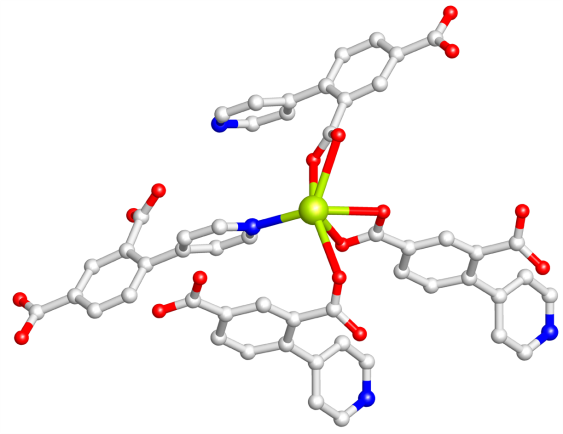

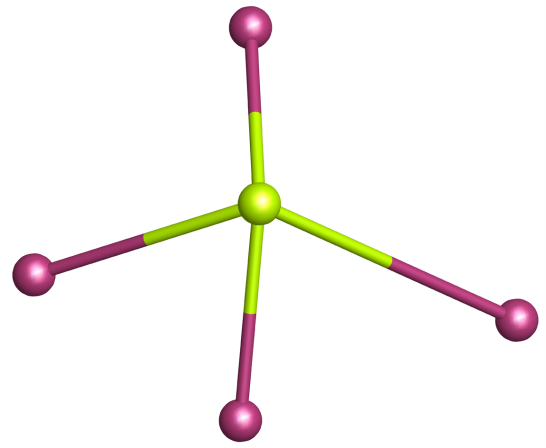
**

**
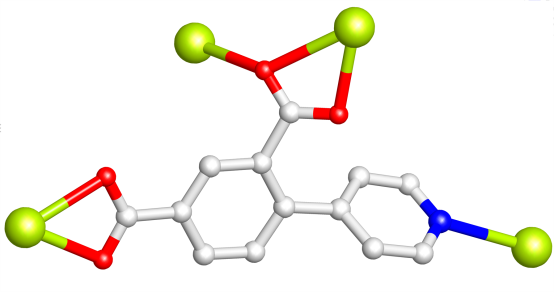

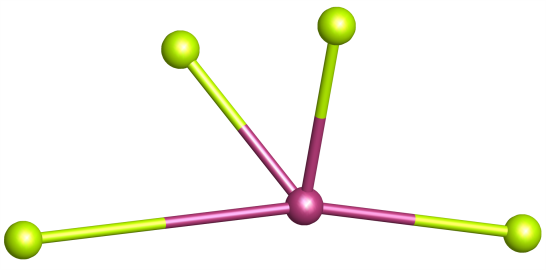
**

(a)

**
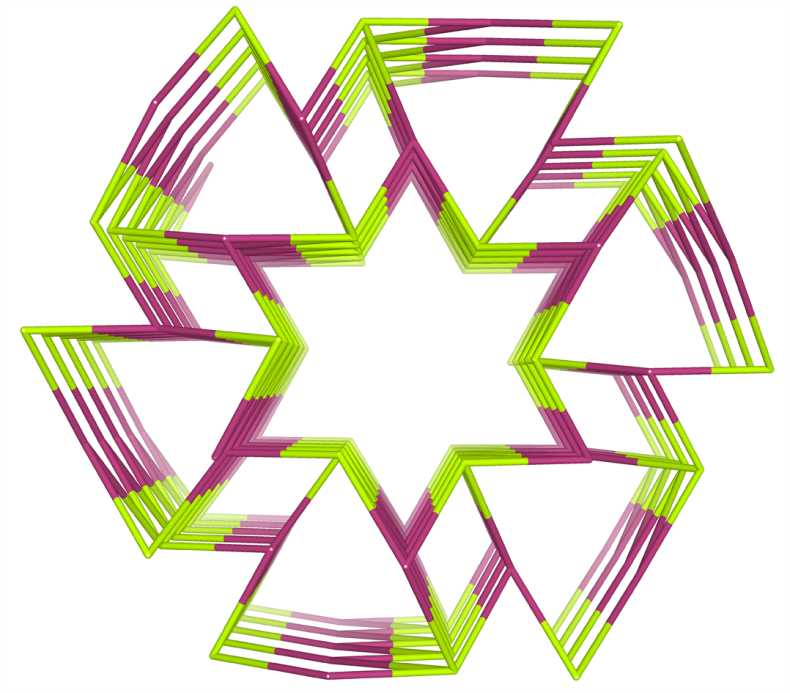
**

(b)

**Figure S8.** (a) View of the 4-connected Pb(II) cation and pyIPA anion. (b) View of (4,4)-connected [6^3^·12^2^][4·6·8^3^] top of **Pb-pyIPA**.

**
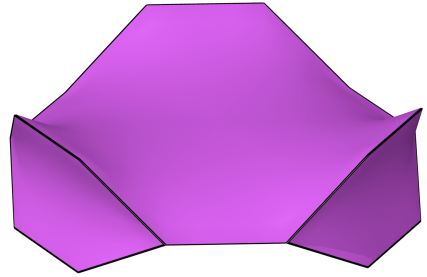

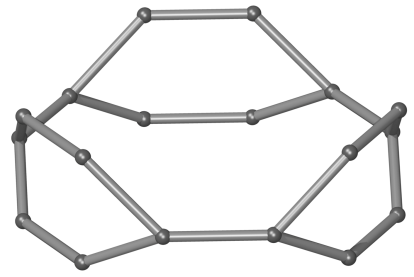

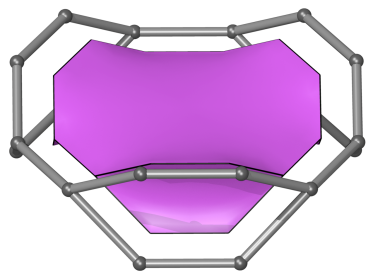
**

(a)

**
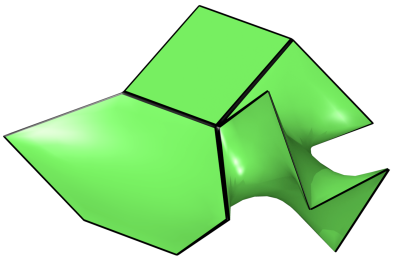

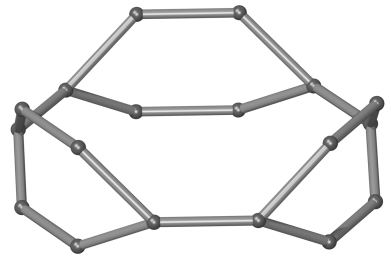

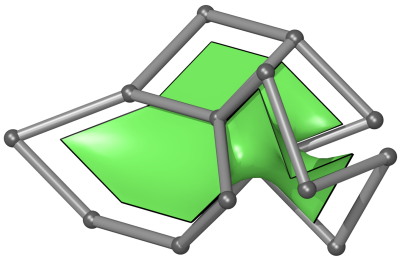
**

(b)

**
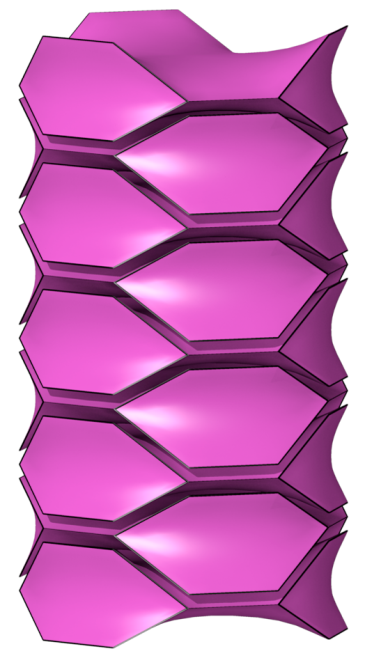

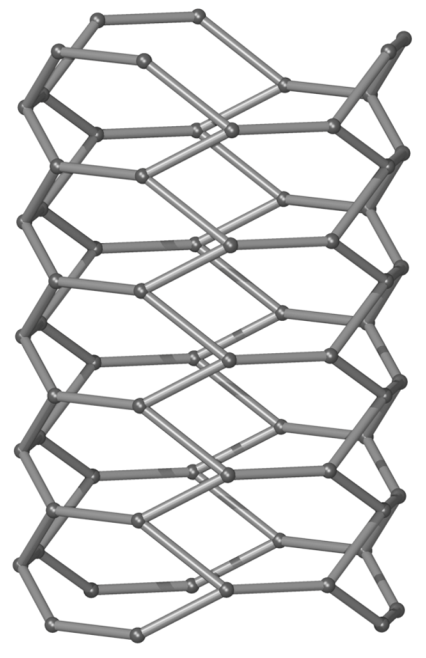
**

(c)

**Figure S9.** Nature tiling representation of the (4,4)-connected net of **Pb-pyIPA**. View of [6^3^.12^2^] (a) and [4.6.8^3^] (b) tiles, (c) Tiling view of 12-number ring channel.


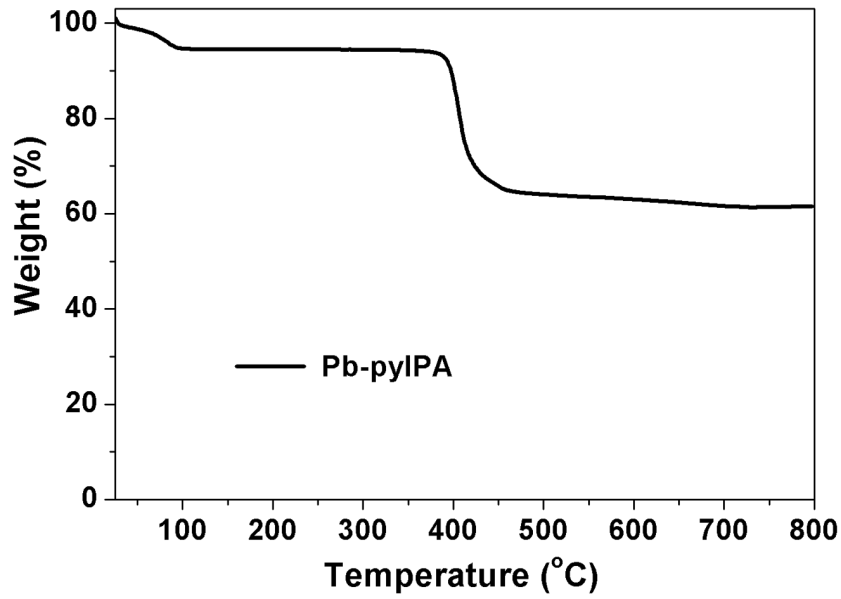


(a)


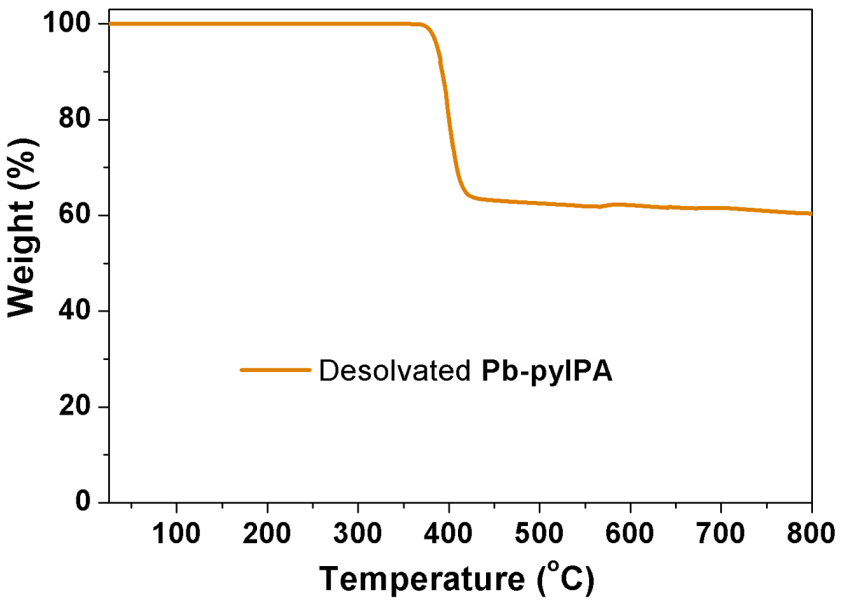


(b)

**Figure S10.** The TGA curve of **Pb-pyIPA** (a) and desolvated **Pb-pyIPA** (b).


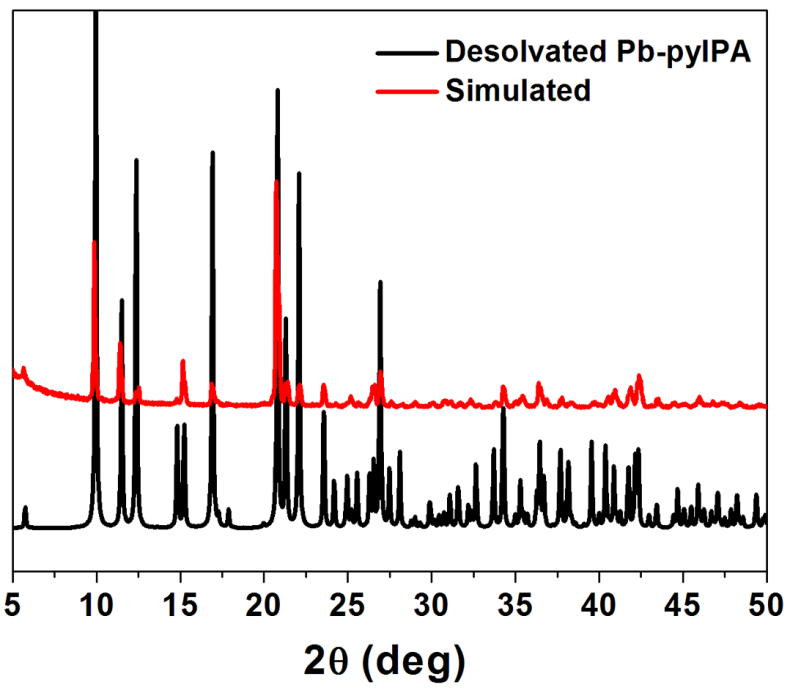


**Figure S11.** The PXRD pattern of desolvated **Pb-pyIPA**.


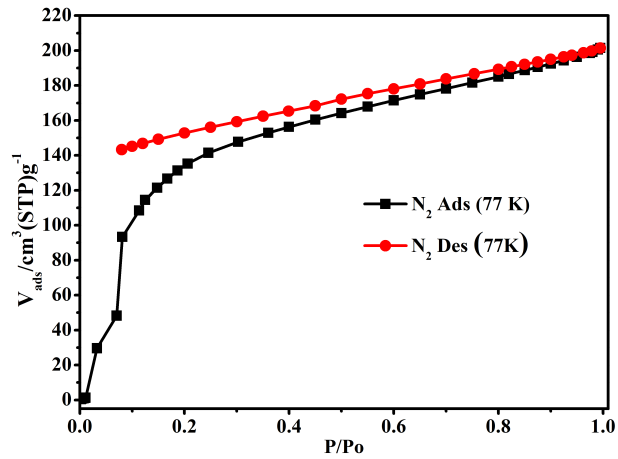

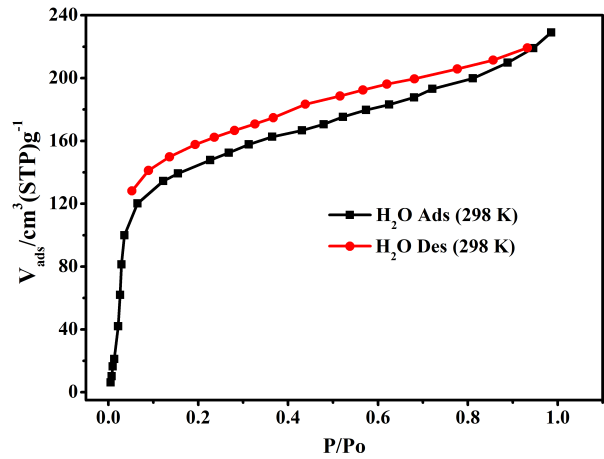


1. (b)

**Figure S12**. (a) The N_2_ adsorption and desorption isotherms of desolvated **Pb-pyIPA** at 77 K; (b) Water adsorption and desorption isotherms of desolvated **Pb-pyIPA** at 298 K.


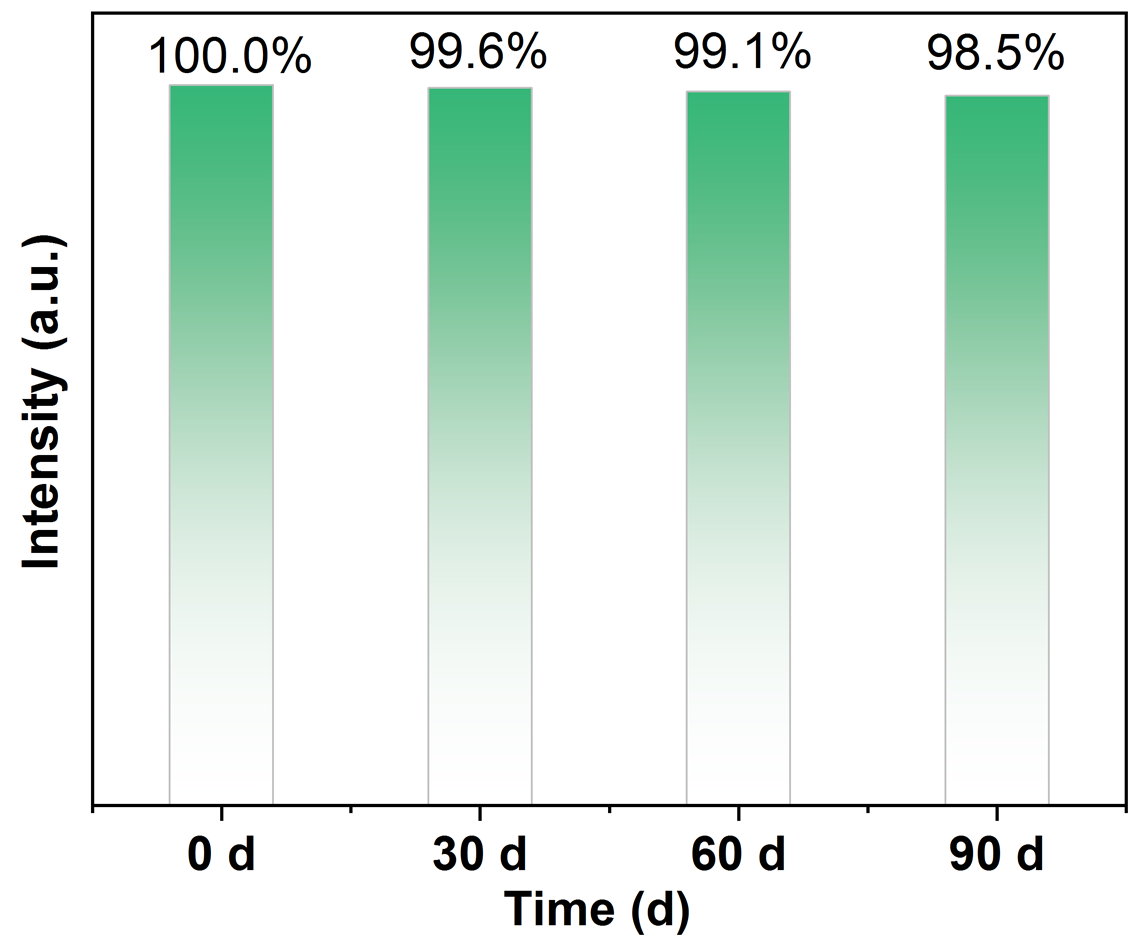


**Figure S13** The intensity of **Pb-pyIPA** measured at the different water-soaking days.


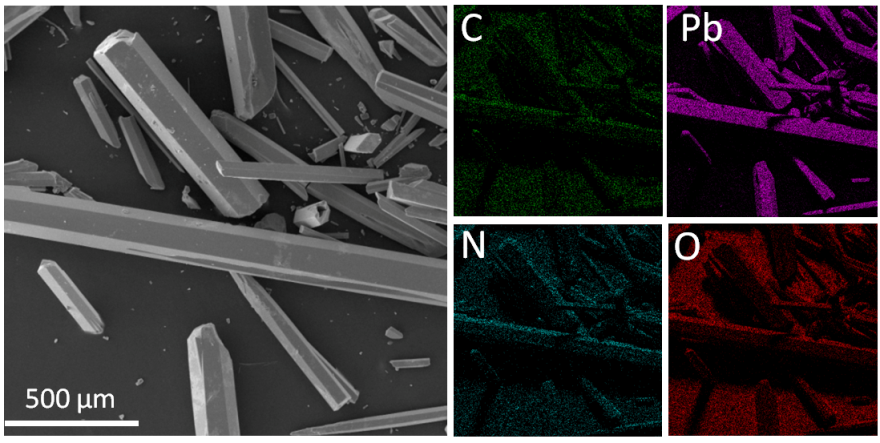


**Figure S14**. SEM and elemental mapping images of **Pb-pyIPA** before soaked in water.


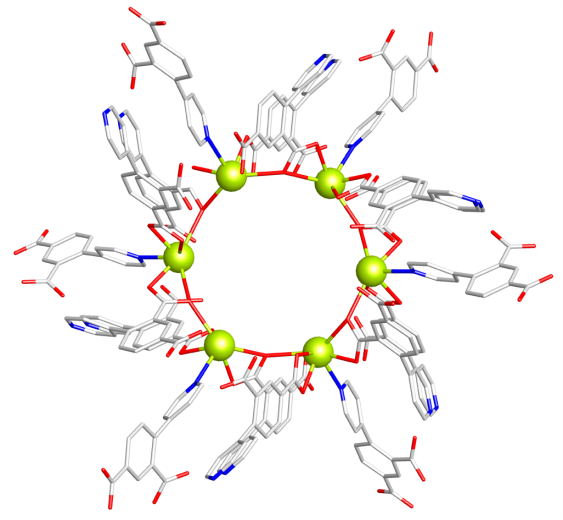

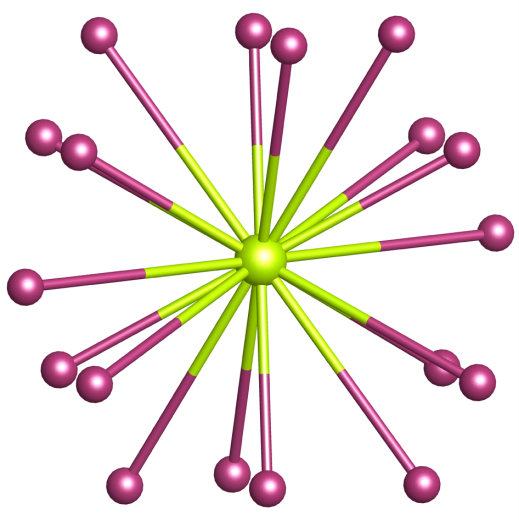


**Figure S15.** View of the {Pb_6_} wheel connected by 18 pyIPA ligands.


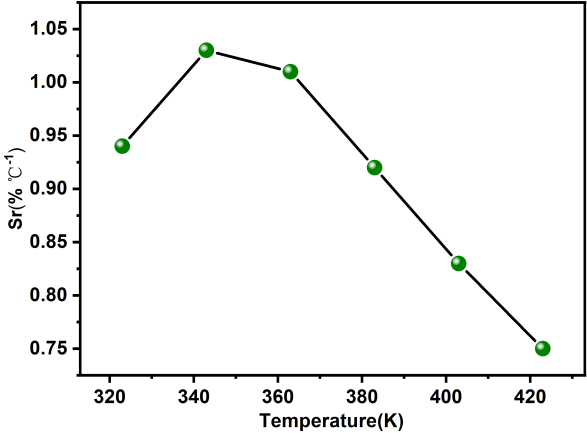

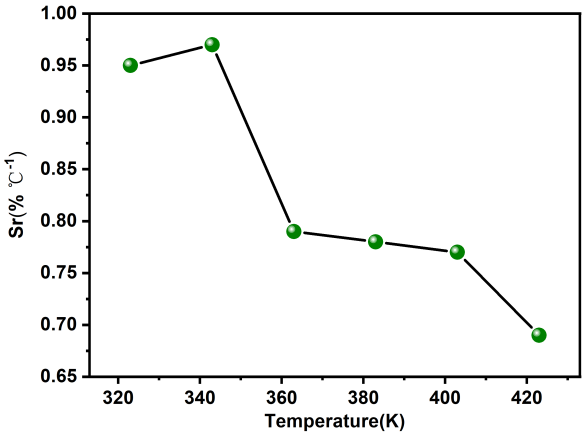


(a) (b)

**Figure S16**. The relative sensitivity (S_r_) based on luminescent intensity (a) and lifetime (b) within the temperature range of 298–423 K.


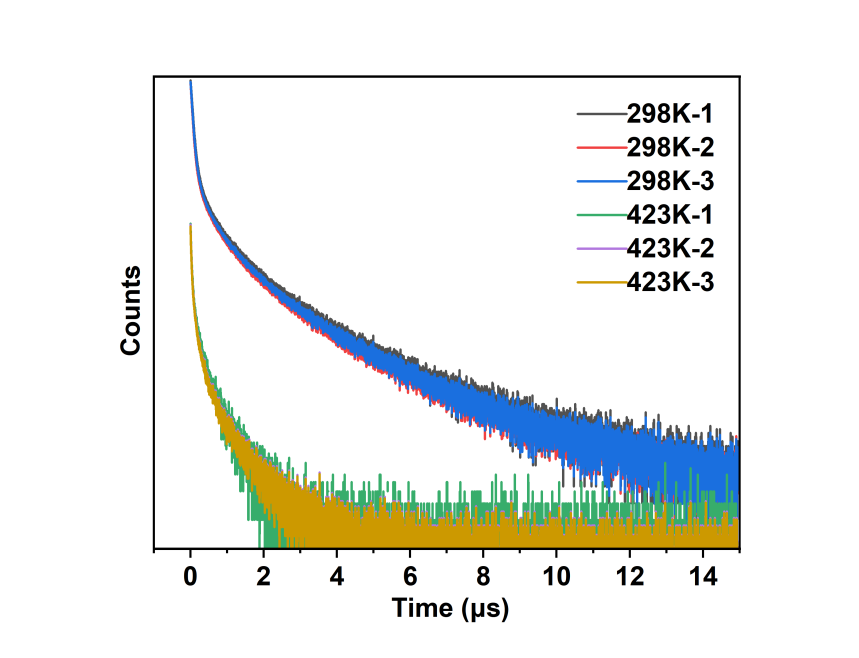


(a)


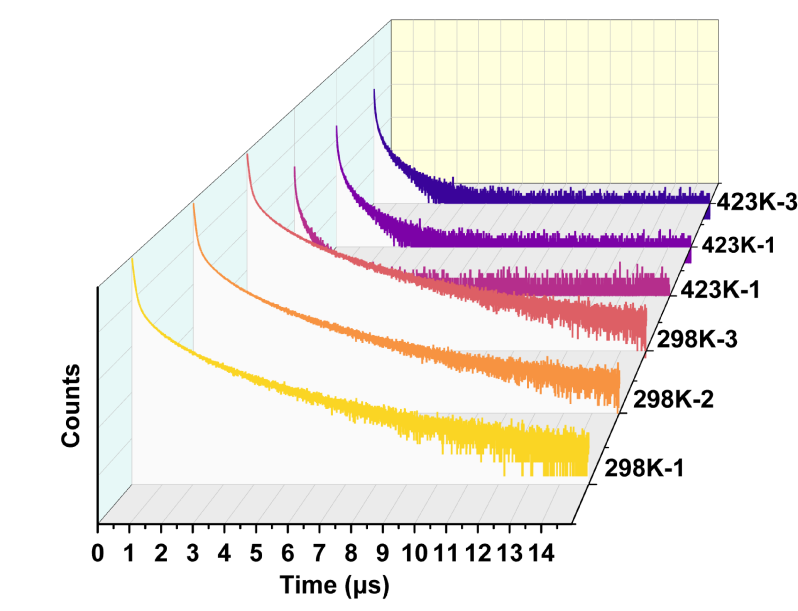


(b)

**Figure S17** The spectral lifetime cycling tests for **Pb-pyIPA**: (a) 2D image display and (b) 3D waterfall mode.


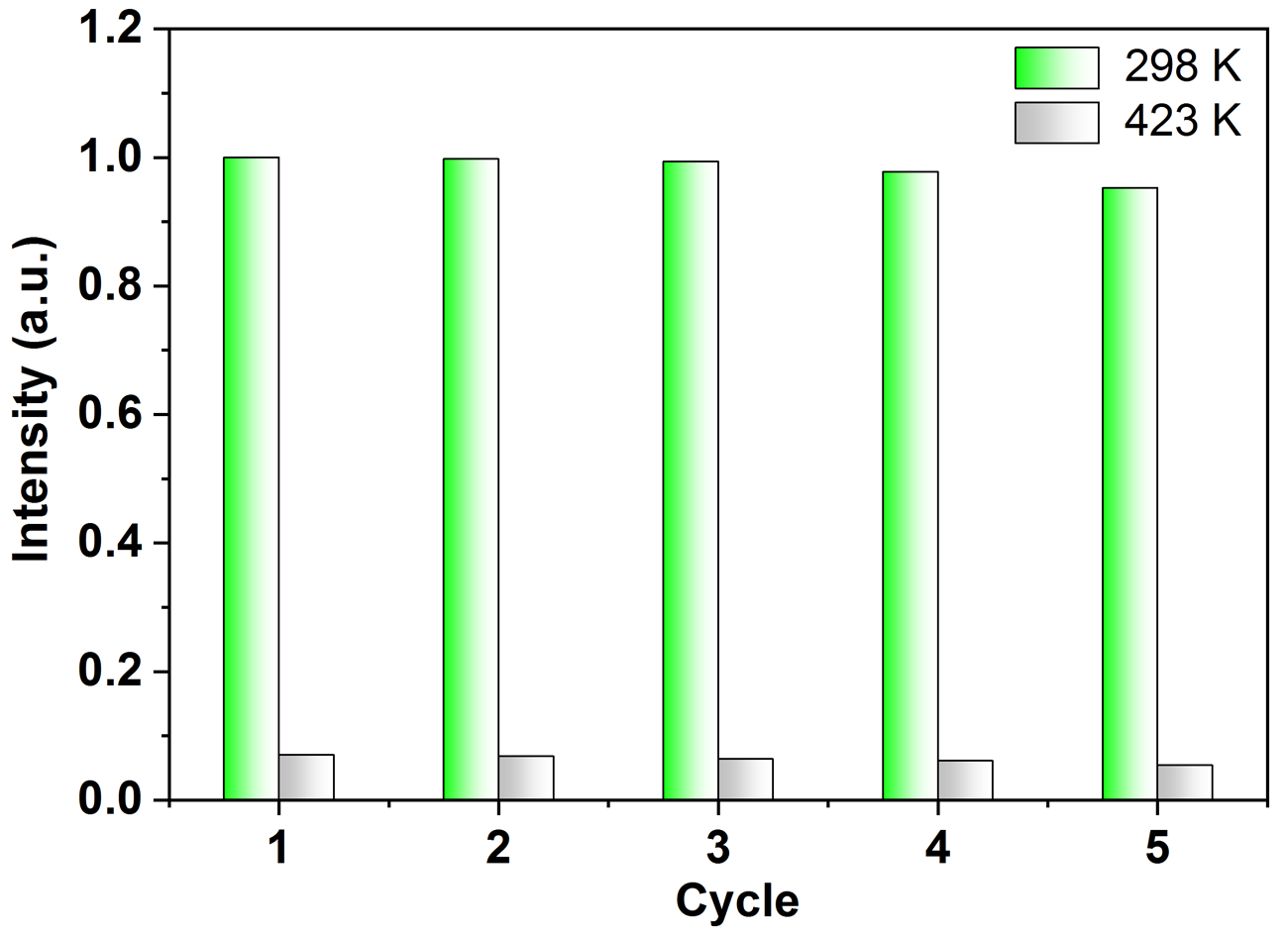


**Figure S18.** The cycle experiments for the emission intensity of **Pb-pyIPA** detected at 298 and 423 k.


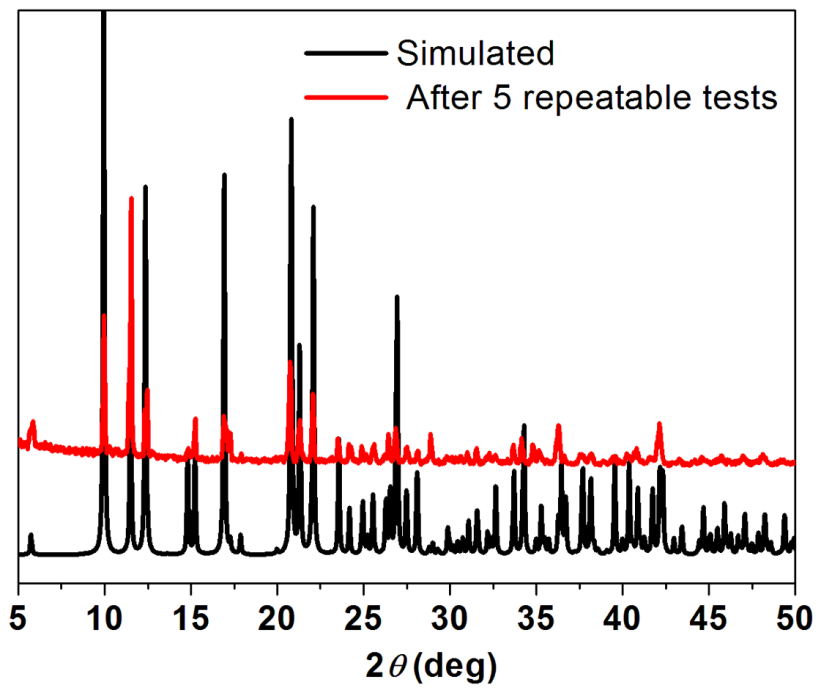


**Figure S19.** PXRD pattern of **Pb-pyIPA** after 5 consecutive cycles between 298 and 423 K.


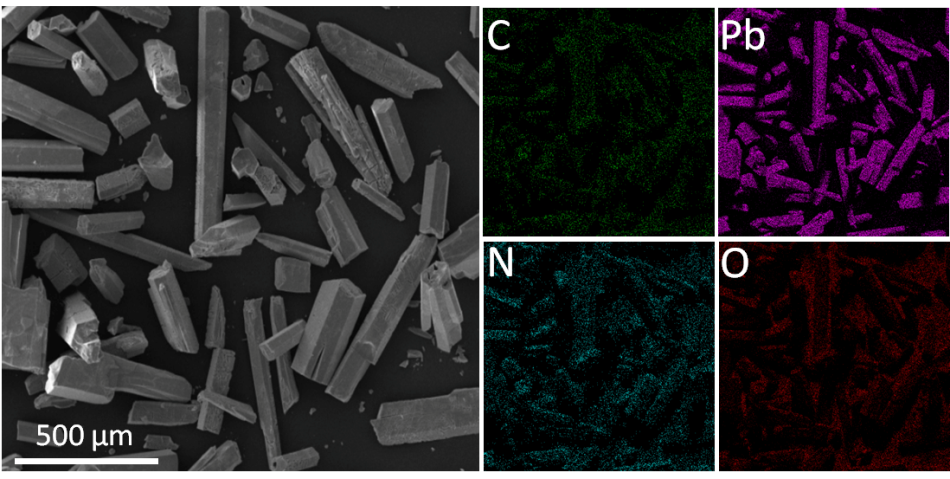


**Figure S20.** SEM and elemental mapping images of **Pb-pyIPA** after 5 consecutive cycles between 298 and 423 K.

**
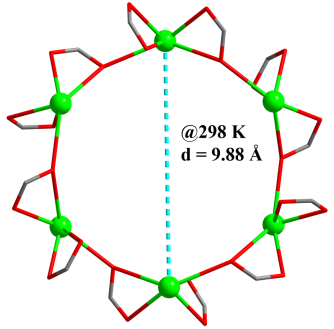

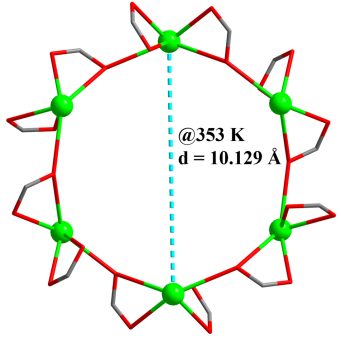
**

(a) (b)

**
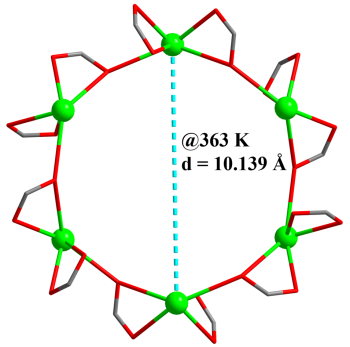

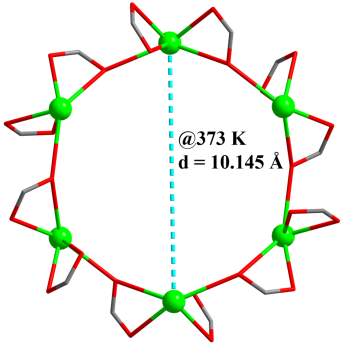
**

(c) (d)

**
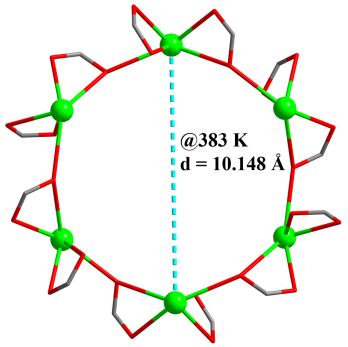

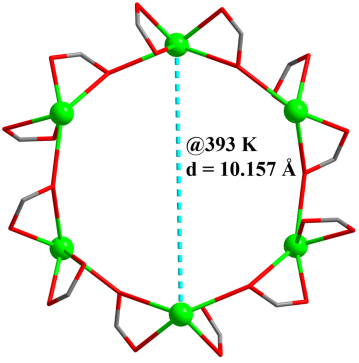
**

(e) (f)

**
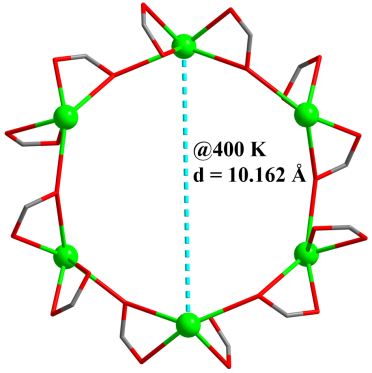
**

(g)

**Figure S21**. The diameters of {Pb_6_} wheel of 1D channel in **Pb-pyIPA** at different temperature.


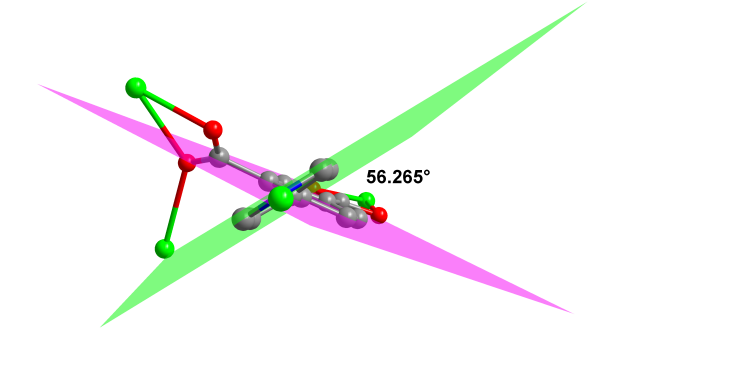

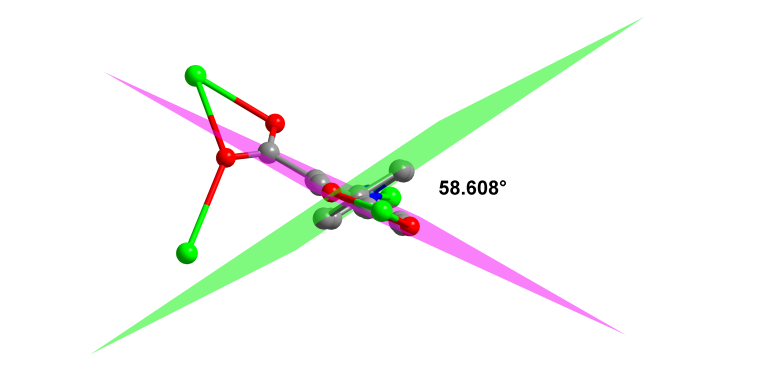


(a) (b)


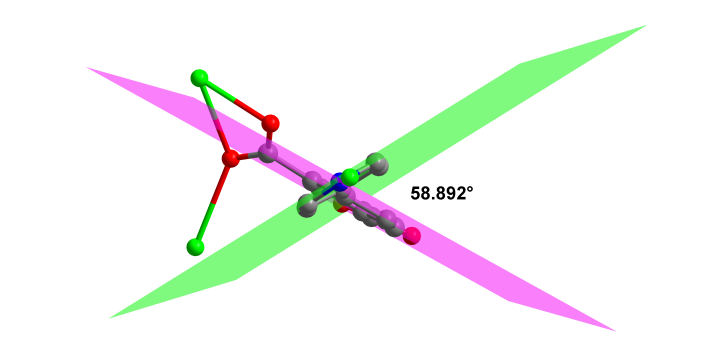

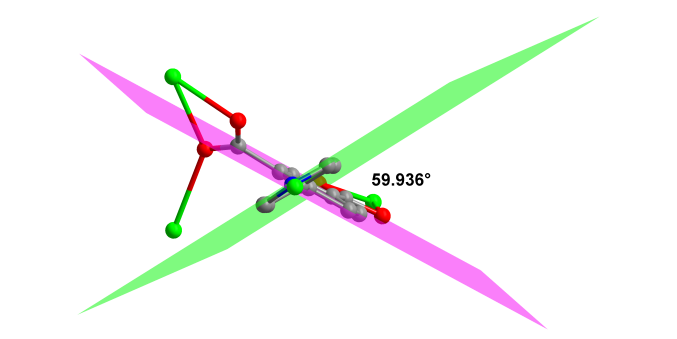


(c) (d)


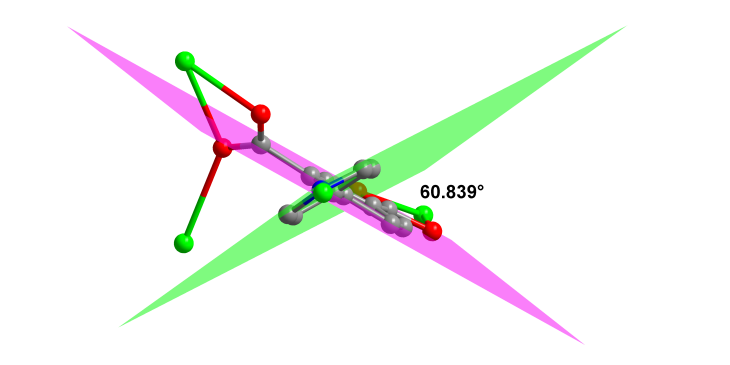

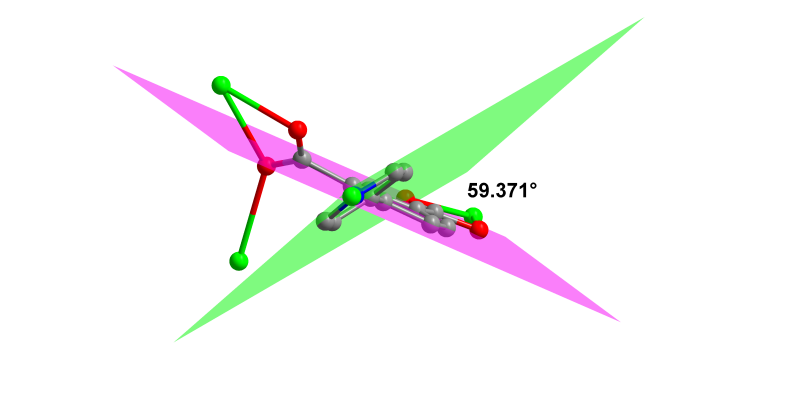


(e) (f)


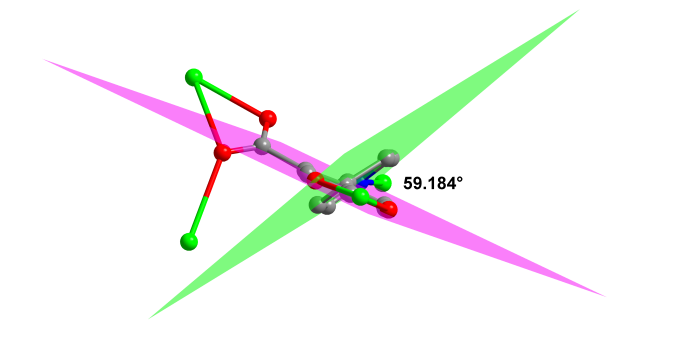


(g)

**Figure S22.** The dihedral angles between the benzene ring and the pyridine ring of the organic ligand in **Pb-pyIPA**: (a) 298 K; (b) 353K; (c) 363 K; (d) 373K; (e) 383 K; (f) 393 K; (g) 400 K.

**3. Supporting Table**

**Table S1.** Crystal data and refinements for **Pb-pyIPA** at different temperature.

| **Pb-pyIPA** | **298 K** | **353 K** | **363 K** | **373 K** | **383 K** | **393 K** | **400 K** |
| --- | --- | --- | --- | --- | --- | --- | --- |
| Formula | C_13_H_7_NO_5.2_Pb | C_13_H_7_NO_4_Pb | C_13_H_7_NO_4_Pb | C_13_H_7_NO_4_Pb | C_13_H_7_NO_4_Pb | C_13_H_7_NO_4_Pb | C_13_H_7_NO_4_Pb |
| Formula weight | 469.4 | 448.39 | 448.39 | 448.39 | 448.39 | 448.39 | 448.39 |
| Crystal system | Trigonal | Trigonal | Trigonal | Trigonal | Trigonal | Trigonal | Trigonal |
| Space group | *R*-3 | *R*-3 | *R*-3 | *R*-3 | *R*-3 | *R*-3 | *R*-3 |
| *a*[Å] | 30.759(5) | 30.8288(6) | 30.8509(6) | 30.8568(6) | 30.8558(14) | 30.8704(7) | 30.8808(7) |
| *c*[Å] | 7.4252(11) | 7.4471(10) | 7.4496(10) | 7.4472(10) | 7.4450(4) | 7.4522(2) | 7.4521(2) |
| *V*[Å^3^] | 6083.9(17) | 6129.6(3) | 6140.4(3) | 6140.8(3) | 6138.6(6) | 6150.3(3) | 6154.4(3) |
| *Z* | 18 | 18 | 18 | 18 | 18 | 18 | 18 |
| D_calcd._[mg·m^-3^] | 2.295 | 2.186 | 2.183 | 2.182 | 2.183 | 2.179 | 2.178 |
| *µ*[mm^-1^] | 12.498 | 24.186 | 24.144 | 24.142 | 24.151 | 24.105 | 24.089 |
| *R*_1_ (*I>2σ* (*I* ))*^a^*  *wR_2_* (*I>2σ*(*I* ))*^b^* | R_1_=0.0380  WR_2_= 0.0815 | R_1_= 0.0301  WR_2_= 0.0719 | R_1_=0.0320  WR_2_=0.0832 | R_1_= 0.0349  WR_2_=0.0839 | R_1_= 0.0579  WR_2_=0.1567 | R_1_= 0.0542  WR_2_=0.1366 | R_1_= 0.0378  WR_2_=0.0848 |

*^a^* *R*_1_=∑||*F*_o_|-|*F*_c_||/∑|*F*_o_|, *wR*_2_=[∑*w*(*F*_o_^2^- *F*_c_^2^)^2^/ ∑*w*(*F*_o_^2^)^2^]^1/2^

**Table S2.** The selected bond lengths (Å) and angles (°) for **Pb-pyIPA**.

| N(1)-Pb(1)#1 | 2.619(7) | O(3)#4-Pb(1)-O(1) | 79.9(2) |
| --- | --- | --- | --- |
| O(1)-Pb(1) | 2.466(6) | O(3)#4-Pb(1)-O(2) | 80.4(2) |
| O(2)-Pb(1) | 2.515(6) | O(1)-Pb(1)-O(2) | 52.3(2) |
| Pb(1)-O(3)#4 | 2.405(6) | O(3)#4-Pb(1)-N(1)#5 | 77.8(2) |
| Pb(1)-N(1)#5 | 2.619(7) | O(1)-Pb(1)-N(1)#5 | 79.0(2) |
| Pb(1)-O(4)#6 | 2.731(6) | O(2)-Pb(1)-N(1)#5 | 129.3(2) |
| Pb(1)-O(4)#4 | 2.752(6) | O(3)#4-Pb(1)-O(4)#6 | 153.2(2) |
| O(1)-Pb(1)-O(4)#4 | 112.64(19) | O(1)-Pb(1)-O(4)#6 | 75.1(2) |
| O(2)-Pb(1)-O(4)#4 | 74.7(2) | O(2)-Pb(1)-O(4)#6 | 76.7(2) |
| N(1)#5-Pb(1)-O(4)#4 | 120.0(2) | N(1)#5-Pb(1)-O(4)#6 | 106.4(2) |
| O(4)#6-Pb(1)-O(4)#4 | 133.6(2) | O(3)#4-Pb(1)-O(4)#4 | 50.01(19) |

Symmetry codes: #1: -y+2/3, x-y+1/3, z+4/3; #2: y, -x+y, -z ; #3: x, y, z+1; #4: x-y, x, -z ; #5: -x+y+1/3, -x+2/3, z-4/3; #6: x, y, z-1.

**Table S3**. Comparison of emission intensity based luminescent thermometers.

| Matrix | T range (Κ) | Sr (% K^−1^) | T_m_(K) | ref |
| --- | --- | --- | --- | --- |
| Tb_5.58_Eu_0.42_-UiO-66 | 255-295 | 1.12 | 255 | [7] |
| **Pb-pyIPA** | 298-423 | 1.03 | 343 | **This work** |
| UCY-17(Eu_0.05_Tb_0.95_)/ABDC | 313-353 | 1.10 | 353 | [8] |
| UCY-17(Eu_0.05_Tb_0.95_)/FBDC | 270-365 | 1.00 | 328 |  |
| UCY-17(Eu_0.05_Tb_0.95_)/NDC | 275-340 | 0.56 | 304 |  |
| UCY-17(Eu_0.05_Tb_0.95_)/BDC | 290-360 | 0.53 | 323 |  |
| DyTPTC-2Me (thermally coupled) | 303-423 | 1.20 | 303 | [9] |
| Eu_0.001_Tb_0.999_(BPDC-0N) | 50-300 | 1.10 | 188 | [10] |
| NaLaTi_2_O_6_ | 253-373 | 1.240 | 253 | [11] |
| NaYF_4_ | 284-344 | 1.080 | 284 | [12] |
| NaYF_4_ | 304-574 | 1.101 | 484 | [13] |
| La_0.915_Eu_0.025_Tb_0.06_ABDC_0.03_ | 10-100 | 0.31 | 34 | [14] |
| Eu_0.15_Tb_0.85_-1,3-BDC | 150-350 | 0.44 | 236 | [15] |
| Eu_0.125_Tb_0.875_-1,3-BDC | 150-350 | 0.40 | 251 |  |
| Eu_0.1_Tb_0.9_-1,3-BDC | 150-350 | 0.31 | 284 |  |
| Eu_0.075_Tb_0.925_-1,3-BDC | 150-350 | 0.24 | 314 |  |
| Eu_0.05_Tb_0.95_-1,3-BDC | 150-350 | 0.20 | 333 |  |
| Eu_0.03_Tb_0.97_-1,3-BDC | 150-350 | 0.19 | 338 |  |
| Eu_0.19_Tb_0.81_PDDI | 313-473 | 0.37 | 473 | [16] |
| SrIn_2_(P_2_O_7_)_2_ | 298-523 | 0.350 | 450 | [17] |
| LaB_3_O_6_ | 298-548 | 0.579 | 423 | [18] |
| Ba_3_Yb(PO_4_)_3_ | 303-603 | 0.650 |  | [19] |
| NaYTiO_4_ | 303-823 | 0.600 | 303 | [20] |
| K_3_YSi_2_O_7_ | 303-663 | 0.705 | 363 | [21] |
| NaYF_4_ | 285-495 | 0.880 | 285 | [22] |

**Table S4.** Comparison of emission lifetime based luminescent thermometers.

| Matrix | T(K) | Sr (% K^−1^) | T_m_(K) | ref |
| --- | --- | --- | --- | --- |
| NaYF_4_ | 304-574 | 1.101 | 405 | [12] |
| **Pb-pyIPA** | **298-423** | 0.97 | 343 | This work |
| Ca_2_MgWO_6_ | 303-573 | 0.110 | 573 | [23] |
| Sr_8_MgL(PO_4)7_ | 298-573 | 0.700 | 573 | [24] |
| Ca_8_ZnLa(PO_4_)_7_ | 298-498 | 0.340 | 498 | [25] |
| SrGa_2_B_2_O_7_ | 293-573 | 0.538 | 293 | [26] |
| MIPr(PO_3_)_4_  Polyphosphate | 298-363 | 0.592 | 363 | [27] |
| Ba_2_GdV_3_O_11_ | 298-573 | 0.146 | 573 | [28] |

**Table S5 The unit cell parameters for Pb-pyIPA at different temperature.**

| T (K) | Cell parameters (Å/°) | Volume (Å^3^) | Diameter (Å) |
| --- | --- | --- | --- |
| 298K | 30.759(5), 30.759(5), 7.4252(11) | 6083.9 | 9.880 |
| 353K | 30.8288(6), 30.8288(6), 7.44714(14) | 6129.6(2) | 10.129 |
| 363K | 30.8509(5); 30.8509(5); 7.4496(14) | 6140.42(19) | 10.139 |
| 373K | 30.8568(6), 30.8568(6), 7.4472(15) | 6140.8(2) | 10.145 |
| 383K | 30.8558(14), 30.8558(14), 7.4450(4) | 6138.6(5) | 10.148 |
| 393K | 30.8704(7), 30.8704(7), 7.4522(2) | 6150.4(3) | 10.157 |
| 400K | 30.8808(7), 30.8808(7), 7.45209(16) | 6154.4(2) | 10.162 |

**4. Supporting References**

1. G. M. Sheldrick, SHELXL-97, Program for Refinement of Crystal Structures, University of Gottingen: Gottingen, Germany **1997**.
2. G. Sheldrick, M. SHELXS, Program for X-ray Crystal Structure Determination, University of Gottingen: Gottingen, Germany **2014**.
3. G. M. Sheldrick, SADABS, Empirical Absorption Correction Program, University of Gottingen: Gottingen, Germany **1997**.
4. B. Delley, *J. Chem. Phys*. 2000, *113*, 7756-7764.
5. S. J. Clark, M. D. Segall, C. J. Pickard, P. J. Hasnip, M. J. Probert, K. Refson, M. C. Payne, *Zeitschrift füer Kristallographie*. 2005, *220*, 567-570.
6. B. Delley, *J. Chem. Phys*. 1990, *92*, 508-517.
7. E. Djanffar, H. A. Bicalho, Z. Ajoyan, A. J. Howarth, H. *J. Mater. Chem. C*. 2024, *12*, 8024-8029.
8. L. K. Komodiki, N. Panagiotou, H. Serier-Brault, A. J. Tasiopoulos, *J. Mater. Chem*. *C*. 2024, *12*, 8684-8696.
9. Z. Li, Q. Wang, K. Yu, W. Cui, Y. He, B. Chen, D. Zhao, *Inorg. Chem*. 2023, *62*, 5652-5659.
10. T. Xia, W. Cao, L. Guan, J. Zhang, F. Jiang, L. Yu, Y. Wan, *Dalton Trans*. 2022, *51*, 5426-5433.
11. Y. Wang, Q. Zhang, C. Yang, Z. Xia, *Adv. Mater*. 2024, *36*, e2401057.
12. M. Meng, T. Zhang, J. Wang, Z. Cheng, Y. Liu, X. Qiao, J. Wen, U. *ACS Appl. Nano Mater*. 2023, *6*, 759-771.
13. A. Zhou, J. Yang, Y. Li, C. Ming, Y. Cai, Y. Pei, *CrystEngComm*. 2024, *26*, 334-340.
14. A. E. Psalti, S. V. Eliseeva, A. Hatzidimitriou, S. Oikonomidis, S. Petoud, T. Lazarides, *J. Am. Chem. Soc*. 2026, *148*, 4020-4031.
15. V. Trannoy, A. N. Carneiro Neto, C. D. S. Brites, L. D. Carlos, H. Serier-Brault, *Adv. Opt. Mater*. 2021, *9*, 2001938.
16. D. Zhao, H. Wang, G. Qian, Synthesis, *CrystEngComm.* 2018, *20*, 7395-7400.
17. Y. Liu, R. Wang, Q. Yang, G. Li, J. Huang, G. Cai, *J. Am. Ceram. Soc*. 2022, *105*, 6184-6195.
18. X. Wu, L. Lou, H. Feng, G. Lv, Q. Wang, D. Zhu, C. Jiang, Z. Mu, *Optik*. 2021, *243*, 167459.
19. Z. Lei, H. Dong, L. Sun, B. Teng, Y. Zou, D. Zhong, *J. Mater. Chem. C*. 2024, *12*, 628-638.
20. Y. Wang, Y. Li, C. Ma, Z. Wen, X. Yuan, Y. Cao, *J. Lumin*. 2022, *248*, 118917.
21. Q. Wang, S. Zhao, J. Wen, X. Huang, C. Wei, Q. Xia, Z. Mu, H. Guo, *Ceram. Int*. 2023, *49*, 41264-41271.
22. M. Meng, T. Zhang, J. Wang, Z. Cheng, J. Yang, X. Qiao, J. Wen, U. Resch-Genger, J. Ou, *Nanotechnology*. 2022, *33*, 455502.
23. Y. Jiang, Y. Tong, S. Chen, W. Zhang, F. Hu, R. Wei, H. Guo, *Chem. Eng. J*. 2021, *413*, 127470.
24. W. Xia, L. Li, P. Yang, F. Ling, Y. Wang, Z. Cao, S. Jiang, G. Xiang, X. Zhou, Y. Hua, *J. Lumin*. 2021, *239*, 118383.
25. L. Li, X. Tang, Z. Wu, Y. Zheng, S. Jiang, X. Tang, G. Xiang, X. Zhou, *J. Alloys Compd*. 2019, *780*, 266-275.
26. X. Liu, S. Shi, K. Yang, L. Chen, D. Deng, S. Xu, *J. Alloys Compd*. 2021, *879*, 160247.
27. S. Gharouel, L. Labrador-Páez, P. Haro-González, K. Horchani-Naifer, M. Férid, *J. Lumin*. 2018, *201*, 372-383.
28. I. Kachou, K. Saidi, U. Ekim, M. Dammak, M. Ersundu, A. Ersundu, *Heliyon*. 2024, *10*, e30062.
